# CDKN1B/p27 is localized in mitochondria and improves respiration-dependent processes in the cardiovascular system—New mode of action for caffeine

**DOI:** 10.1371/journal.pbio.2004408

**Published:** 2018-06-21

**Authors:** Niloofar Ale-Agha, Christine Goy, Philipp Jakobs, Ioakim Spyridopoulos, Stefanie Gonnissen, Nadine Dyballa-Rukes, Karin Aufenvenne, Florian von Ameln, Mark Zurek, Tim Spannbrucker, Olaf Eckermann, Sascha Jakob, Simone Gorressen, Marcel Abrams, Maria Grandoch, Jens W. Fischer, Karl Köhrer, René Deenen, Klaus Unfried, Joachim Altschmied, Judith Haendeler

**Affiliations:** 1 Heisenberg-group—Environmentally-induced Cardiovascular Degeneration, Medical Faculty, HHU Duesseldorf and IUF-Leibniz Research Institute for Environmental Medicine, Duesseldorf, Germany; 2 Institute of Genetic Medicine, Newcastle University, Newcastle upon Tyne, United Kingdom; 3 Core Unit Biosafety Level 2 Laboratory, IUF-Leibniz Research Institute for Environmental Medicine, Duesseldorf, Germany; 4 Environmentally-induced Skin and Lung Aging, IUF-Leibniz Research Institute for Environmental Medicine, Duesseldorf, Germany; 5 Institute for Pharmacology and Clinical Pharmacology, Medical Faculty, HHU Duesseldorf, Duesseldorf, Germany; 6 Biological and Medical Research Center (BMFZ), HHU, Duesseldorf, Germany; University of Pittsburgh, United States of America

## Abstract

We show that the cyclin-dependent kinase inhibitor 1B (CDKN1B)/p27, previously known as a cell cycle inhibitor, is also localized within mitochondria. The migratory capacity of endothelial cells, which need intact mitochondria, is completely dependent on mitochondrial p27. Mitochondrial p27 improves mitochondrial membrane potential, increases adenosine triphosphate (ATP) content, and is required for the promigratory effect of caffeine. Domain mapping of p27 revealed that the N-terminus and C-terminus are required for those improvements. Further analysis of those regions revealed that the translocation of p27 into the mitochondria and its promigratory activity depend on serine 10 and threonine 187. In addition, mitochondrial p27 protects cardiomyocytes against apoptosis. Moreover, mitochondrial p27 is necessary and sufficient for cardiac myofibroblast differentiation. In addition, p27 deficiency and aging decrease respiration in heart mitochondria. Caffeine does not increase respiration in p27-deficient animals, whereas aged mice display improvement after 10 days of caffeine in drinking water. Moreover, caffeine induces transcriptome changes in a p27-dependent manner, affecting mostly genes relevant for mitochondrial processes. Caffeine also reduces infarct size after myocardial infarction in prediabetic mice and increases mitochondrial p27. Our data characterize mitochondrial p27 as a common denominator that improves mitochondria-dependent processes and define an increase in mitochondrial p27 as a new mode of action of caffeine.

## Author summary

The protein p27 is a nuclear cell cycle inhibitor that can be shuttled to the cytoplasm to inactivate its inhibitory role, and this mechanism is thought to be used by cancer cells to unlock cell cycle arrest. Recent reports, however, have shown that p27 has other roles independent of cell cycle regulation, and it was observed that p27 mutant mice had increased mortality to myocardial infarction. Here, we have analyzed the potential role of p27 in the major cell types of the heart and shown that it is also present in mitochondria, the cellular powerhouses, where it fulfils important functions. We find that p27 is required for migration of endothelial cells by enhancing mitochondrial functions and that caffeine concentrations reached after consumption of 4 cups of coffee induce its translocation into mitochondria. Moreover, we observe that mitochondrial p27 protects heart muscle cells from cell death and is necessary for the conversion of fibroblasts into mechanically strong, contractile myofibroblasts, a process critical after myocardial infarction. Molecularly, we show that p27 is essential for caffeine-induced gene expression changes that mainly affect mitochondria and for mitochondrial respiration. We conclude that mitochondrial p27 improves mitochondria-dependent processes in heart cells and that physiological concentrations of caffeine have a protective effect.

## Introduction

The cyclin-dependent kinase inhibitor 1B (CDKN1B), also known as p27, was initially discovered as a nuclear-localized cell cycle inhibitor [1]. Previous data demonstrating that p27 can be exported to the cytoplasm [2,3] were considered as a mechanism to inactivate the cell cycle inhibitory effects of p27 in the nucleus and to allow human cancer cells to escape cell cycle arrest. However, McAllister and colleagues demonstrated that nonnuclear p27 is required for migration of fibroblasts, since p27-deficient mouse embryonic fibroblasts failed to migrate, while reconstitution with p27 rescued the motility defect. Its promigratory effect was independent of its cell cycle arrest functions but rather required serine 10 phosphorylation–dependent nuclear export and a C-terminal scatter domain [4]. Moreover, it was suggested that knockout of a cell cycle inhibitor like p27 could be beneficial in the experimental setup of myocardial infarction. This was based on the reasoning that myocardial infarction leads to loss of cells in the heart and that enhanced proliferation of cells in p27-deficient mice may result in smaller infarct size and reduced mortality; however, exactly the opposite was observed [5,6]. Moreover, over the last several years, it has become evident that functional mitochondria, not only in cardiomyocytes but also in endothelial cells [7,8] and in cardiac fibroblasts [9], are required for proper functionality of those cells and are essential for protective actions in cardiovascular diseases.

Furthermore, in recent years, a number of cohort studies have convincingly demonstrated that habitual coffee consumption is associated with a lower risk of developing type 2 diabetes [10,11]. Coffee consumption was inversely correlated with total as well as cause-specific mortality, such as heart disease, respiratory disease, stroke, and diabetes, whereas no relation or a positive correlation was found with cancer-related deaths [12,13]. In addition, several studies have shown that consumption of caffeinated coffee is associated with lower risk for coronary heart disease mortality, specifically in older subjects [14,15]. Finally, the beneficial effect of caffeine appeared to be dose-dependent, as coffee consumption of 4 cups or more per day resulted in a further reduced risk for adverse events when compared to lower coffee consumption. We established previously that 4 cups of coffee lead to a serum concentration of approximately 30 μM caffeine in humans [8]. Therefore, mechanisms explaining the protective effects of caffeine should be attributed to serum concentrations of less than 100 μM. Over decades, the effects of caffeine have been ascribed to its antagonist activity on adenosine receptors, inhibition of phosphodiesterases (PDEs), and elevated intracellular calcium levels. Since a raise of intracellular calcium in different cell types requires at least 500 μM caffeine, which in humans would result in lethal intoxication [16–18], effects on intracellular calcium can be excluded as a potential mechanism. Similarly, inhibition of PDEs by caffeine requires concentrations of 250 μM or higher, depending on the isoforms investigated [19,20]. Studies regarding the responses to activation or inhibition of adenosine receptors in the cardiovascular system are controversial. Activation of the adenosine 2A receptor has beneficial effects in the infarcted porcine myocardium [21], whereas blockade of the adenosine 2A receptor reduces cardiac reactive oxygen species production and expression of NADPH oxidase 2 in the heart [22]. Thus, it remains unclear whether unspecific inhibition of adenosine receptors or PDEs by caffeine could explain the protective effects of coffee consumption. Importantly, we demonstrated that caffeine in physiologically relevant concentrations improves the functional capacity of endothelial cells ex vivo and in vivo in a mitochondria-dependent manner [8].

Given the described protective role of caffeine and its association with mitochondria, we hypothesized that a common denominator exists in endothelial cells, cardiomyocytes, and cardiac fibroblasts that improves the mitochondria-dependent functionalities of those cells ex vivo and in vivo. Since the role of nonnuclear p27 in nontumor cells was never examined in detail, we investigated whether p27 is present in the mitochondria and is indeed required to improve mitochondria-dependent functionalities and whether the protective caffeine effects are causally related to mitochondrial p27, which would present a new mode of action for caffeine, explaining its protective function in the cardiovascular system.

## Results

### Mitochondrial p27 is indispensable for functional improvement of endothelial cells

Physiologically relevant concentrations of caffeine, which have beneficial cardiovascular effects, have been attributed to 4 or more cups of daily coffee consumption. Four cups of coffee lead to a serum caffeine concentration of approximately 30 μM in humans [8]. Since 4 or more cups of coffee seem to have a beneficial effect, we used 50 μM caffeine in all cellular studies presented here, as well as concentrations of caffeine in the drinking water of mice, which result in approximately 30–50 μM in the serum of the animals [8]. To assess a potential involvement of adenosine receptors in the caffeine-mediated effects, we first investigated the impact of caffeine on endothelial cell migration, as a measure for functional capacity, in the presence of adenosine receptor 2A and 2B blockers SCH442416 and GS6201, respectively. Neither inhibition of adenosine receptor 2A nor 2B changed the ability of 50 μM caffeine to induce migration in human primary endothelial cells (S1 Fig). Moreover, caffeine did not change phosphorylation of PDEs 4A and 5A, respectively (S2 Fig), which is in accordance with the literature that caffeine concentrations higher than 250 μM are needed to modulate activity of those enzymes and thus to change intracellular cyclic nucleotide levels [19,20].

**Fig 1:**
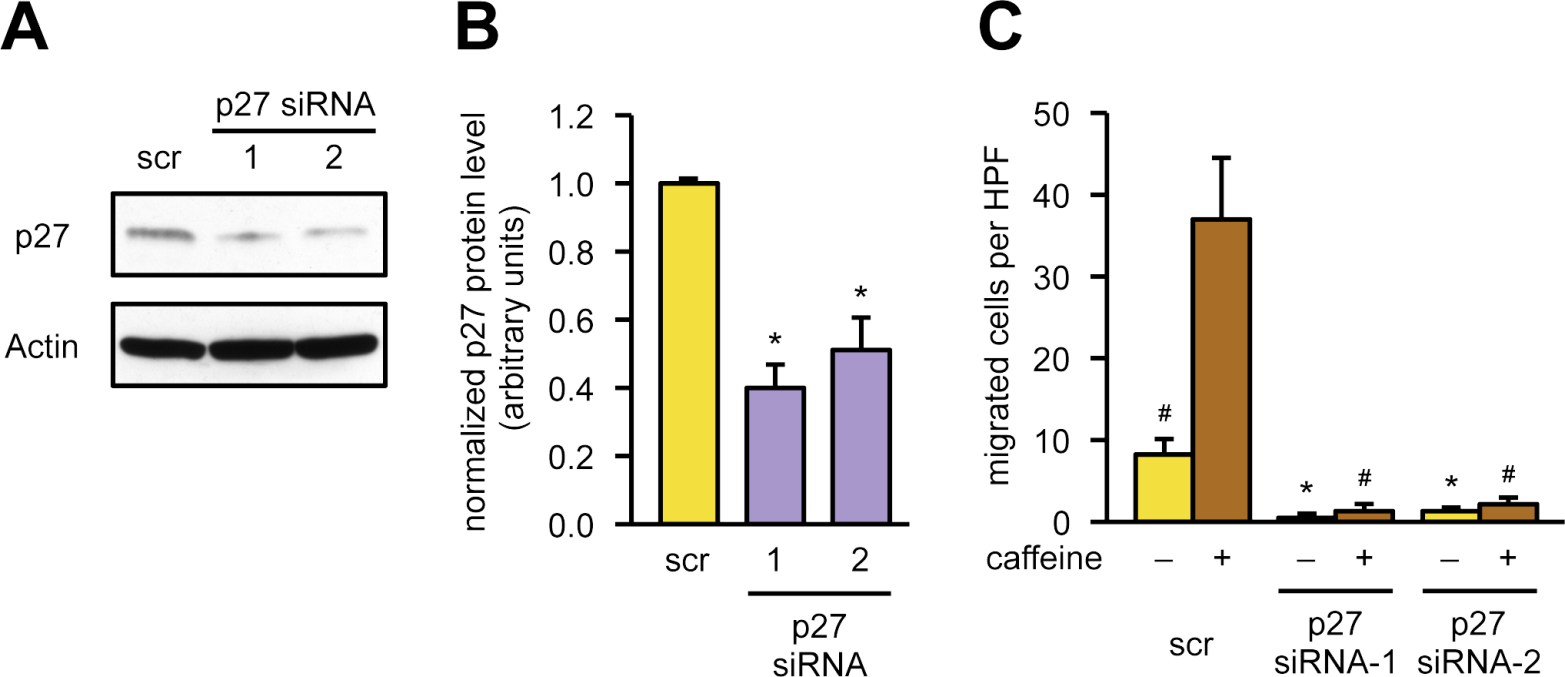
p27 is required for endothelial cell migration. **(A, B)** p27 was knocked down in endothelial cells by transfection with 2 different siRNAs targeting the p27 mRNA (“p27 siRNA-1,” “p27 siRNA-2”) or a scrambled siRNA (“scr”) as control, and p27 levels were determined by immunoblot. **(A)** Representative immunoblots, Actin served as loading control. **(B)** Knockdown efficiency was determined by semiquantitative analysis of immunoblots. Data are mean ± SEM, *n* = 5, *p < 0.05 versus scr (one-way ANOVA). **(C)** Endothelial cells were transfected with the same siRNAs as before, a wound was set 48 hours after transfection, and the cells were treated with 50 μM caffeine for another 18 hours or left untreated. Migratory capacity was assessed by counting cells migrated into the wound using Image J. Data are mean ± SEM, *n* = 5, *p < 0.05 versus scr −caffeine, ^#^*p* < 0.05 versus scr +caffeine (one-way ANOVA). Underlying data are provided in S1 Data. HPF, high power field; siRNA, small interfering RNA.

McAllister and colleagues showed that p27 is necessary for migration of HepG2 cells and embryonic fibroblasts. Furthermore, its promigratory effect was independent of its cell cycle arrest functions but rather required serine 10 phosphorylation–dependent nuclear export and a C-terminal scatter domain [4]. Therefore, we down-regulated p27 with 2 different small interfering RNAs (siRNAs; Figs 1A, 1B and S3) and determined first the effect on cell viability (siRNA1: 106.9 ± 11.9%; siRNA2: 134.6 ± 21.4% of scrambled control, *n* = 5, means ± SEM, not significant) as well as on cellular and mitochondrial morphology (S4 Fig). Since transfection of p27-specific siRNAs affected neither cell viability nor morphology, we next investigated the effect on endothelial cell migration. Basal as well as caffeine-induced migration was completely blunted upon knockdown of p27 (Fig 1C). These data demonstrate that primary human endothelial cells require p27 for migration.

**Fig 2:**
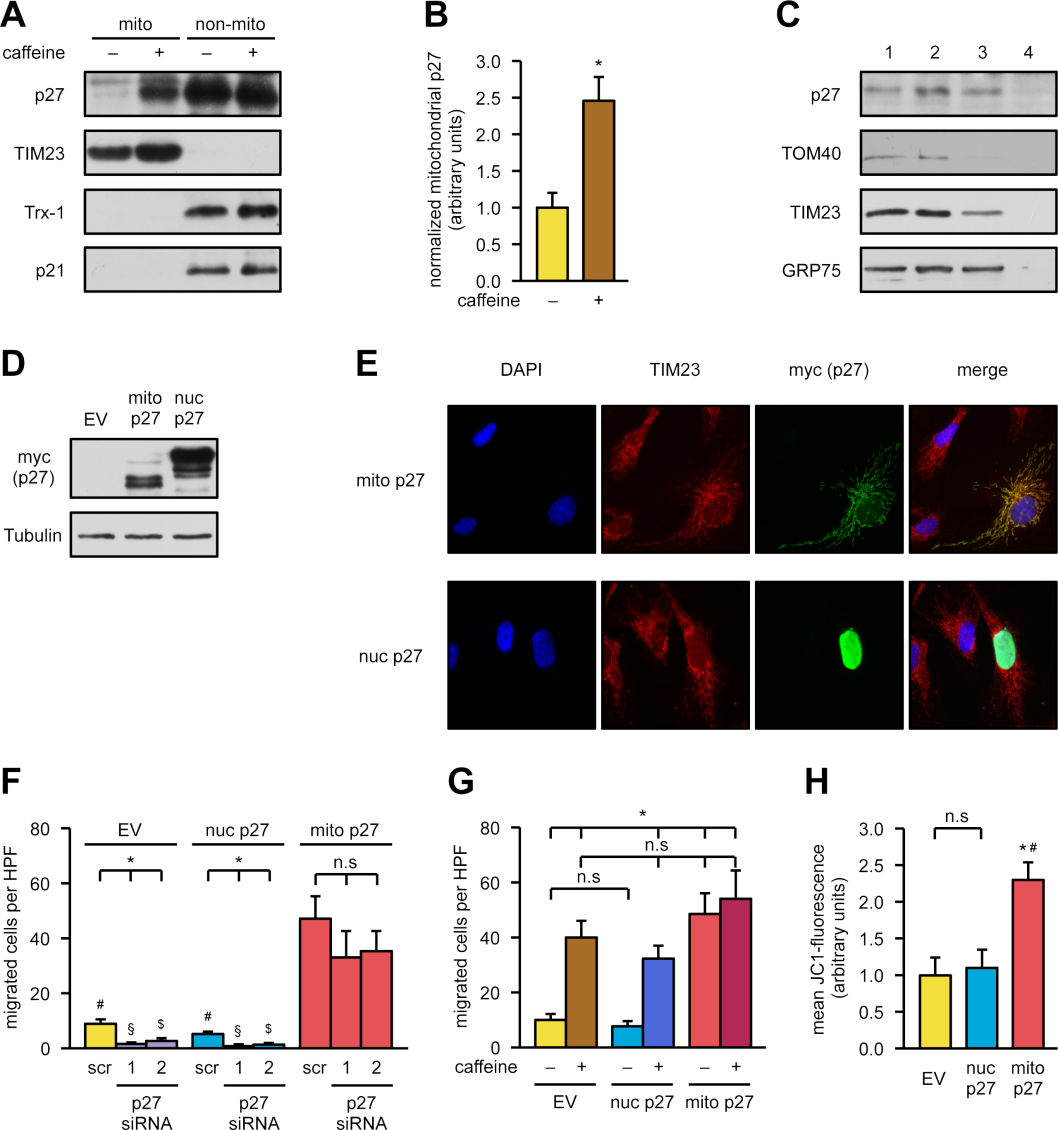
Mitochondrial p27 is sufficient to induce endothelial cell migration. **(A, B)** Endothelial cells were treated with 50 μM caffeine for 18 hours, and mitochondrial (“mito”) and nonmitochondrial (“non-mito”) fractions were separated. p27 and the closely related p21 protein were detected by immunoblot; TIM23 and Trx-1 served as purity controls for the fractions. **(A)** Representative immunoblots. **(B)** Semiquantitative analysis of mitochondrial p27 normalized to TIM23. Data are mean ± SEM, *n* = 6, *p < 0.05 (two-tailed unpaired *t* test). **(C)** Proteinase K digestion of mitochondria. The different digestion conditions yield intact mitochondria (1), mitochondria stripped of attached proteins (2), and mitoplasts (3); 4 denotes complete digestion. p27 and marker proteins for the outer (TOM40) or inner (TIM23) mitochondrial membrane and the mitochondrial matrix (GRP75) were detected by immunoblot. **(D, E)** Endothelial cells were transfected with an empty vector (“EV”) or expression vectors for nuclear (“nuc p27”) or mitochondrial p27 (“mito p27”). Expression and localization of the organelle-targeted p27 proteins were analyzed by immunoblot and immunofluorescence. **(D)** Representative immunoblot, Tubulin served as loading control. Because of the presence of a trimeric nuclear localization signal at the C-terminus, the nuclear-targeted protein has a larger molecular weight. **(E)** Representative immunostainings: nuclei were visualized with DAPI (blue), mitochondria by staining for TIM23 (red), and the targeted p27 variants by staining for the myc epitope (“myc (p27),” green). Merge shows an overlay of all fluorescence channels. **(F)** Endothelial cells were transfected with the siRNAs used in Fig 1. Forty-eight hours later, cells were transfected with an empty vector (“EV”) or the expression vectors for nuclear (“nuc p27”) or mitochondrial p27 (“mito p27”). Three hours later, a wound was set. Migratory capacity was assessed 18 hours later by counting cells migrated into the wound using Image J. Data are mean ± SEM, *n* = 5: p27, p27 siRNA-1/EV, p27 siRNA-2/EV, p27 siRNA-1/nuc p27 siRNA-1/mito p27; *n* = 6: all others, *p < 0.05 versus corresponding scr, ^#^*p* < 0.05 versus scr/mito p27, ^§^*p* < 0.05 versus p27 siRNA-1/mito p27, ^$^*p* < 0.05 versus p27 siRNA-2/mito p27 (one-way ANOVA). **(G)** Endothelial cells were transfected with an empty vector (“EV”) or expression vectors for nuclear (“nuc p27”) or mitochondrial p27 (“mito p27”). Three hours later, a wound was set, and cells were treated with 50 μM caffeine for 18 hours or left untreated. Migratory capacity was assessed by counting cells migrated into the wound using Image J. Data are mean ± SEM, *n* = 5–7, *p < 0.05 versus EV −caffeine (Mann-Whitney pairwise comparison with Bonferroni-corrected *p*-values). **(H)** Endothelial cells were transfected with an empty vector (“EV”) or expression vectors for nuclear (“nuc p27”) or mitochondrial p27 (“mito p27”). Twenty-four hours after transfection, the mitochondrial membrane potential was measured with JC1 using flow cytometry. Data are mean ± SEM, *n* = 5, *p < 0.05 versus EV, ^#^*p* < 0.05 versus nuc p27 (one-way ANOVA). Underlying data are provided in S1 Data. DAPI, 4′,6-diamidino-2-phenylindole; HPF, high power field; n.s., not significant; TIM23, translocase of inner mitochondrial membrane 23; TOM40, translocase of outer mitochondrial membrane 40; Trx-1, thioredoxin-1.

Since functional mitochondria are necessary for endothelial cell migration [8] and protein translocation to these organelles is a major determinant of their functional capacity [23], we wanted to establish a causal link between mitochondria and p27. Therefore, we investigated whether p27 is localized in mitochondria. As shown by immunoblots following biochemical separation, a fraction of p27 is localized in mitochondria. The purity of the mitochondrial preparations was confirmed by detection of the nonmitochondrial protein thioredoxin-1 (Trx-1) and the mitochondrial translocase of inner mitochondrial membrane 23 (TIM23), respectively. As an additional control, we also detected the cyclin-dependent kinase inhibitor 1A (CDKN1A), also known as p21, a member of the same protein family. As demonstrated in Fig 2A, p21 is not localized in the mitochondria, and caffeine does not affect the protein levels. Moreover, treatment with caffeine significantly increased mitochondrial p27 (Figs 2A, 2B and S5). To further verify that p27 is truly localized in the mitochondria and not simply attached to these organelles, we performed a proteinase K digest of isolated mitochondria. As demonstrated in Fig 2C, p27 is indeed localized within the mitochondria. Digestion of the outer mitochondrial membrane with proteinase K in hypotonic buffer results in mitoplasts, mitochondria stripped of their outer membrane, leaving only the inner mitochondrial membrane and the matrix. The immunoblot analysis confirmed loss of translocase of outer mitochondrial membrane 40 (TOM40) but revealed inner mitochondrial membrane proteins like TIM23 and matrix proteins like the mitochondrial heat shock protein 70, also called heat shock protein 70 kDa protein 9 (HSPA9; or 75 KDa glucose-regulated protein [GRP75]), respectively, and also p27 (Fig 2C). To causally link migration to mitochondrial p27, we cloned targeted variants of p27, which are exclusively localized in the nucleus or mitochondria, and expressed them in endothelial cells. Overexpression of nuclear- as well as mitochondrially targeted p27 revealed comparable expression levels (Fig 2D). Moreover, mitochondrially targeted p27 is exclusively found in the mitochondria; conversely, nuclear-targeted p27 could only be detected in the nucleus (Fig 2E). We then established a rescue experiment in which endogenous p27 was first knocked down by siRNAs, followed by overexpression of nuclear- or mitochondrially targeted p27. Only mitochondrially targeted p27 rescued the migratory defect induced by knockdown of p27, whereas nuclear-targeted p27 did not improve the migratory capacity (Fig 2F). Next, we investigated whether induction of migration by mitochondrial p27 can be further increased by caffeine. Therefore, we overexpressed mitochondrial p27 in endothelial cells, treated the cells with caffeine, and measured migratory capacity; nuclear-targeted p27 and an empty vector served as controls. Caffeine increased migratory capacity in cells transfected with the empty vector or expressing nuclear p27 (Fig 2G). Without caffeine, only mitochondrially targeted p27 induced migration of endothelial cells; however, the combination of caffeine and mitochondrial p27 did not show any additive effects. Thus, caffeine and mitochondrial p27 either share a common promigratory pathway, or each individual stimulus already induced maximal migratory capacity in these cells (Fig 2G). To evaluate whether mitochondrial p27, but not nuclear p27, improves mitochondrial parameters, we measured mitochondrial membrane potential in endothelial cells overexpressing mitochondrial p27 or nuclear p27, respectively. Only mitochondrially targeted p27 significantly enhanced mitochondrial membrane potential (Fig 2H).

### The N- and C-terminus of mitochondrial p27 with serine 10 and threonine 187 are required for migratory capacity of endothelial cells

**Fig 3:**
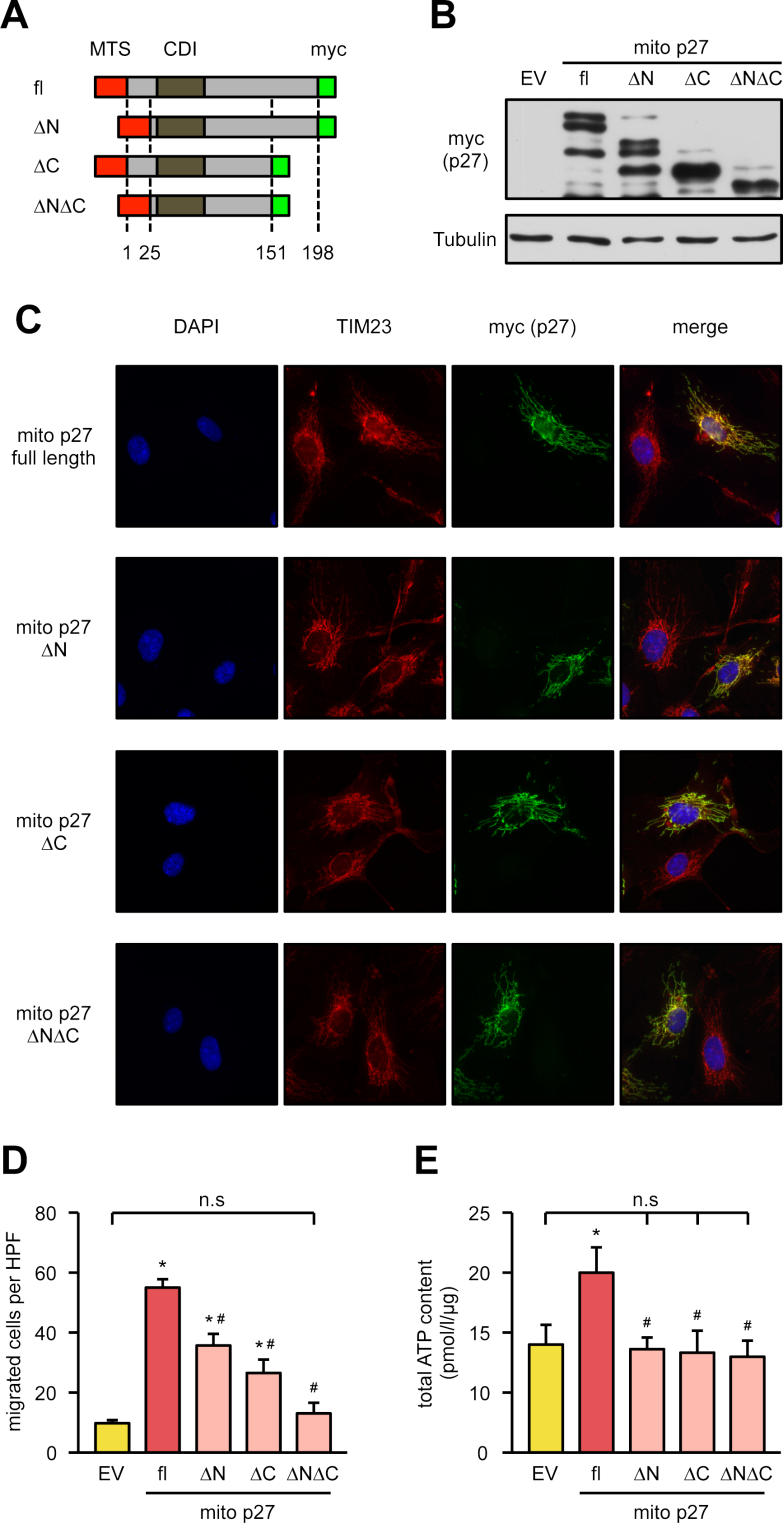
The N- and C-terminus of p27 are required for endothelial cell migration and ATP content. **(A)** Schematic representation of mitochondrially targeted p27 deletion mutants lacking the N-terminus (“ΔN”), the C-terminus (“ΔC”), or both (“ΔNΔC”). The full-length protein (“fl”) and all mutants contain an N-terminal mitochondrial targeting sequence (“MTS,” red) and a C-terminal myc tag (green). Numbers indicate the deletion endpoints within p27. **(B-E)** Endothelial cells were transfected with an empty vector (“EV”) or expression vectors for the mitochondrially targeted p27 mutants depicted in (A). **(B, C)** Expression and localization of the mitochondrially targeted mutant p27 proteins were analyzed by immunoblot and immunofluorescence. **(B)** Representative immunoblot, tubulin served as loading control. **(C)** Representative immunostainings: nuclei were visualized with DAPI (blue), mitochondria by staining for TIM23 (red), and the targeted p27 mutants by staining for the myc epitope (“myc (p27),” green). Merge shows an overlay of all fluorescence channels. **(D)** Migratory capacity was measured in a scratch wound assay by counting cells migrated into the wound using Image J. Data are mean ± SEM, *n* = 6, *p < 0.05 versus EV, ^#^*p* < 0.05 versus fl mito p27 (one-way ANOVA). **(E)** ATP content was measured with a luminometric assay. Data are mean ± SEM, *n* = 5, *p < 0.05 versus EV, ^#^*p* < 0.05 versus fl mito p27 (one-way ANOVA). Underlying data are provided in S1 Data. ATP, adenosine triphosphate; CDI, cyclin-dependent kinase inhibitor; DAPI, 4′,6-diamidino-2-phenylindole; HPF, high power field; n.s., not significant; TIM23, translocase of inner mitochondrial membrane 23.

Given the novelty of our findings, we wanted to understand which domains in p27 could be responsible for its effects on cell migration and mitochondrial functions. Subcellular distribution of p27 was described to be regulated by phosphorylation of at least 4 phosphorylation sites at serine 10, threonine 157, threonine 187, and threonine 198, all of which have been suggested to be important for nonnuclear localization. The role of phosphorylation at these sites is discussed controversially, as it could differ dependent on cell and tumor type or organ system. Nevertheless, as nuclear p27 cannot compensate for the loss of migratory capacity after knockdown of the endogenous protein, it is suggestive that these sites may play a role in migration, which depends on mitochondria. Therefore, we decided to generate p27 mutants with a mitochondrial targeting sequence, in which either the N-terminus with serine 10 (ΔN, amino acids [aas] 25–198 retained), the C-terminus with the other phosphorylation sites (ΔC, aas 1–151 retained), or both (ΔN/ΔC, aas 25–151 retained) were deleted, leaving the cyclin-dependent kinase inhibitor (CDI) domain intact in every construct (Fig 3A). We first confirmed by immunoblotting that all of the mutants are expressed at comparable levels (Fig 3B). Then, we confirmed that the mutants are exclusively localized in the mitochondria (Fig 3C). Next, we examined their impact on functional capacity of human primary endothelial cells by measuring migration and adenosine triphosphate (ATP) content. Cells overexpressing the ΔN or the ΔC mutant showed only a reduced migratory capacity compared to full-length p27, whereas the ΔN/ΔC mutant completely lost the ability to induce migration (Fig 3D). Full-length p27 increased mitochondrial ATP content (Fig 3E). In contrast, this was not observed in cells overexpressing any of the p27 deletion mutants (Fig 3E). These data demonstrate that both the N- and C-terminus of p27 are required for the functional capacity of endothelial cells.

**Fig 4:**
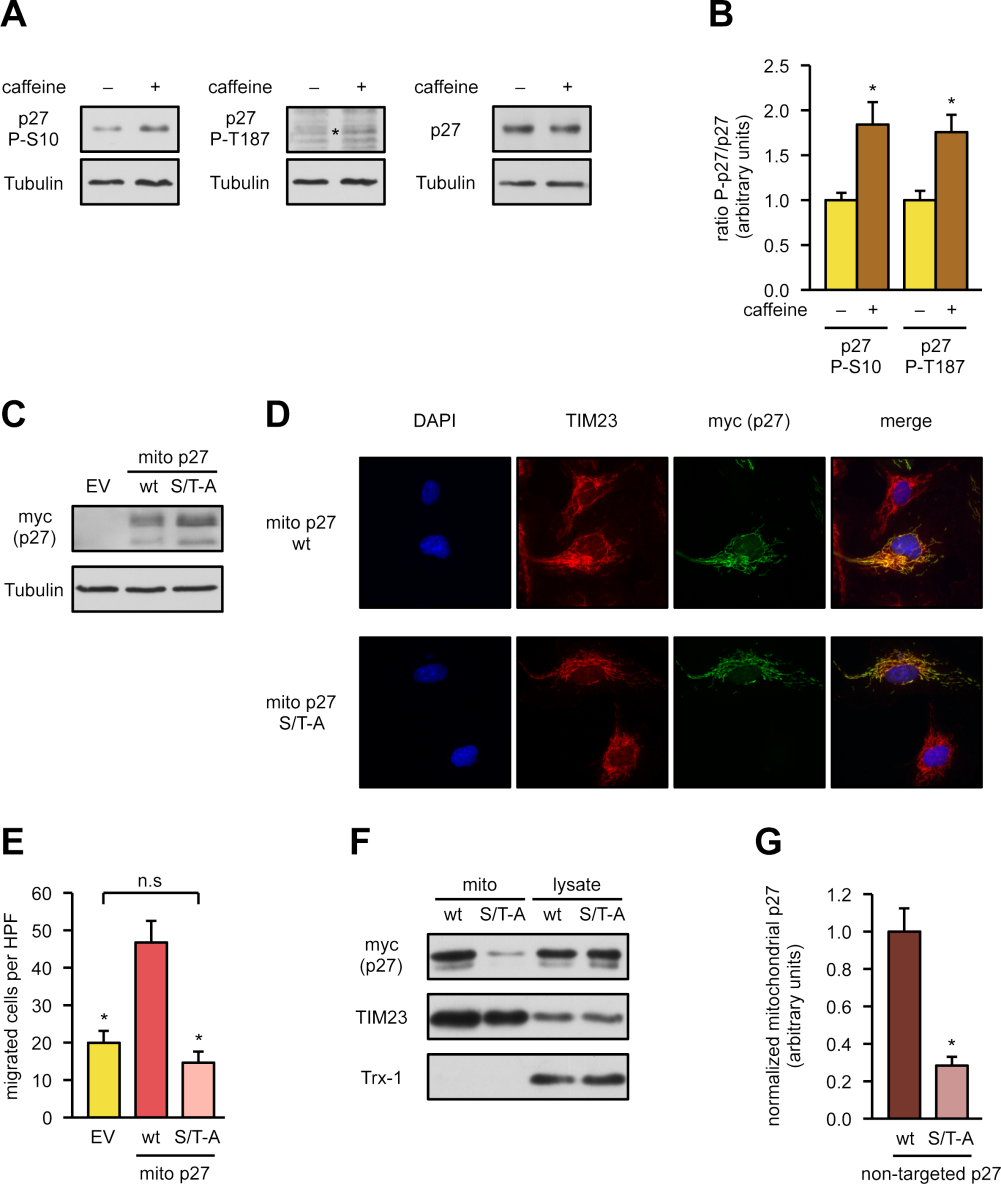
Serine 10 and threonine 187 of p27 are required for endothelial cell migration and mitochondrial import. **(A, B)** Endothelial cells were treated with caffeine for 18 hours or left untreated, and phosphorylation of serine 10 (“p27 P-S10”) and threonine 187 (“p27 P-T187), as well as total p27 (“p27”), were detected by immunoblot. **(A)** Representative immunoblots with the corresponding loading control (Tubulin) below the respective immunoblot. The asterisk denotes p27 phosphorylated on threonine 187. **(B)** Semiquantitative analyses of the ratio of phosphorylated p27 to total p27 for both phosphorylation events. Data are mean ± SEM, *n* = 7: p27 P-S10, *n* = 6: p27 P-T187, *p < 0.05 (two-tailed unpaired *t* test). **(C, D)** Endothelial cells were transfected with an empty vector (“EV”) and expression vectors for mitochondrially targeted p27 (“mito p27 wt”) or a mutant in which serine 10 and threonine 187 were replaced by alanine (“mito p27 S/T-A”). Expression and localization of the corresponding proteins were analyzed by immunoblot and immunofluorescence. **(C)** Representative immunoblot, tubulin served as loading control. **(D)** Representative immunostainings: nuclei were visualized with DAPI (blue), mitochondria by staining for TIM23 (red), and the targeted p27 mutants by staining for the myc epitope (“myc (p27),” green). Merge shows an overlay of all fluorescence channels. **(E)** Endothelial cells were transfected as in (C), a wound was set, and migratory capacity was assessed by counting cells migrated into the wound using Image J. Data are mean ± SEM, *n* = 5, *p < 0.05 versus mito p27 wt (one-way ANOVA). **(F, G)** Endothelial cells were transfected with expression vectors for p27 wild type or the corresponding S/T-A mutant, both without a mitochondrial targeting sequence. Mitochondrial fractions (“mito”) were prepared, and the expressed proteins were detected by immunoblot. **(F)** Representative immunoblots: the p27 proteins were detected with an anti-myc antibody (“myc (p27)”), TIM23 served as a loading control, and Trx-1 as purity control for the mitochondrial fractions. Analysis of total cell lysates (“lysate”) ensures similar expression levels. **(G)** Semiquantitative analysis of mitochondrial p27 normalized to TIM23. Data are mean ± SEM, *n* = 5, *p < 0.05 versus p27 wt (two-tailed unpaired *t* test). Underlying data are provided in S1 Data. DAPI, 4′,6-diamidino-2-phenylindole; HPF, high power field; n.s., not significant; TIM23, translocase of inner mitochondrial membrane 23; Trx-1, thioredoxin-1.

To further narrow down the aas relevant for mitochondrial p27, we focused on serine 10 and threonine 187 as the more likely candidates for phosphorylation because threonine 157 and 198 have been described as relevant for p27/cyclin D1/cyclin-dependent kinase 4 (CDK4) complex assembly and as such for cell cycle regulation [24]. Moreover, for the cardiovascular system, it has been shown that p27 phosphorylation at serine 10 is reduced in murine and human atherosclerotic arteries and that prevention of this phosphorylation aggravates atherosclerosis independent of cell proliferation [25]. Phosphorylation at threonine 187 has been demonstrated to result in proteasomal degradation of p27 in several cancer cells [26]; however, in the cardiovascular system, loss of this phosphorylation did not affect aortic p27 protein levels [27]. Therefore, we first examined whether caffeine induces phosphorylation of p27 at serine 10 and threonine 187. Indeed, 50 μM caffeine increased phosphorylation at both sites by approximately 2-fold (Fig 4A and 4B). Since both the N- and C-terminus are required for the functional capacity of p27 in endothelial cells (Fig 3D and 3E), we generated a mitochondrially targeted, nonphosphorylatable p27(S10A/T187A) double mutant and measured the impact on migratory capacity compared to mitochondrially targeted full-length p27. Besides comparable expression levels between p27 wild type and the mutant (Fig 4C), immunostainings confirmed the mitochondrial localization (Fig 4D). Strikingly, overexpression of this mutant did not induce migration in endothelial cells (Fig 4E). Thus, serine 10 and threonine 187, at least in the mitochondrial fraction of p27, are required for migratory capacity. To elucidate whether these two residues are also necessary for the import of p27 into the mitochondria, we generated an analogous but untargeted p27(S10A/T187A) mutant. Following expression of this variant and the corresponding wild-type protein in endothelial cells, their protein levels in mitochondrial fractions were measured. Interestingly, the ability of p27(S10A/T187A) to become imported into the mitochondria was severely restricted (Fig 4F and 4G), suggesting that the amino acids, which are critical for migratory capacity, are also involved in the translocation into the mitochondria.

### Caffeine effects in the heart are linked to mitochondrial p27

**Fig 5:**
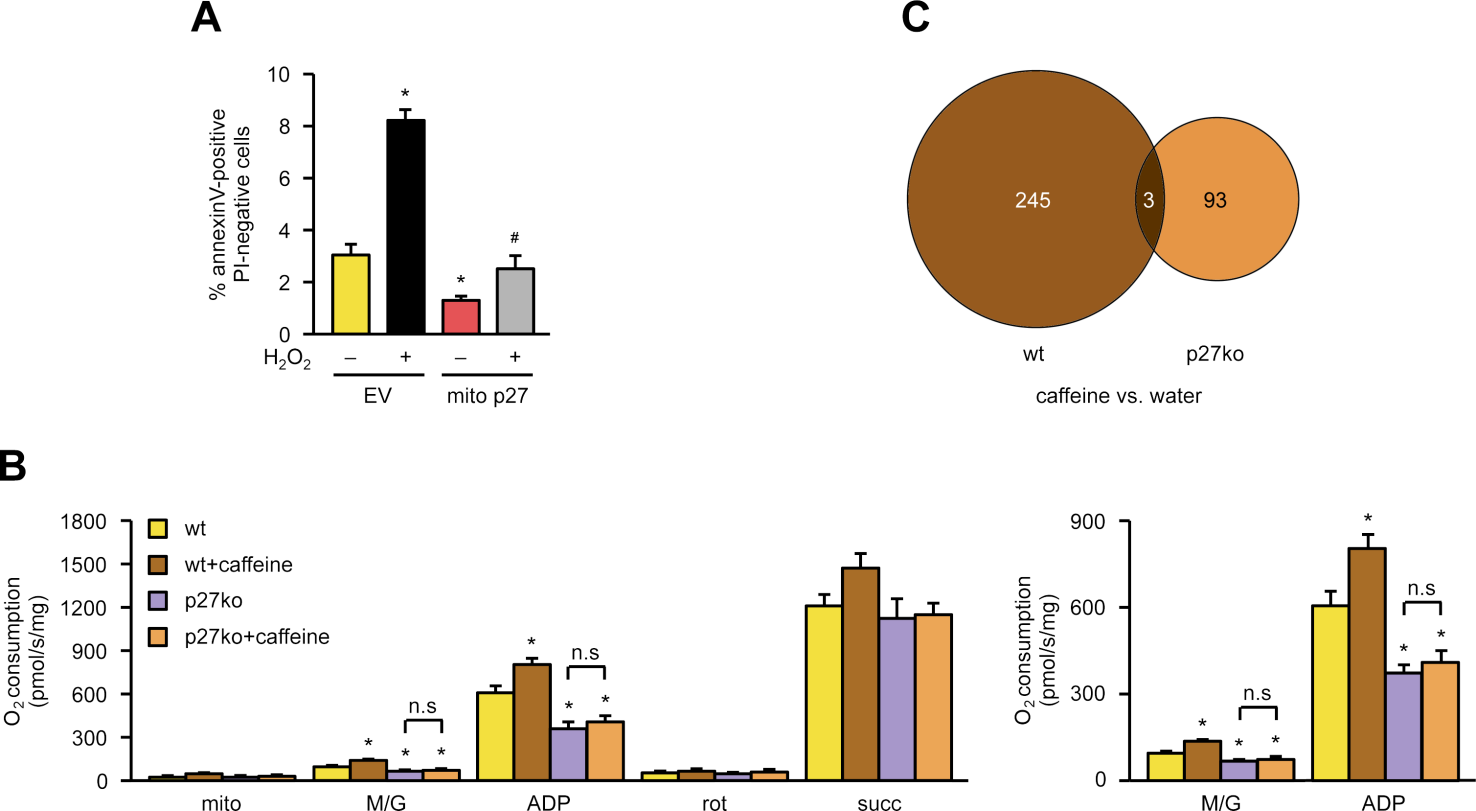
Caffeine effects in the heart depend on p27. **(A)** The mouse cardiomyocyte cell line HL-1 was lentivirally transduced with an empty vector (“EV”) or an expression vector for mitochondrially targeted p27 (“mito p27”) and treated with 500 μM H_2_O_2_ for 48 hours. Apoptosis was measured as annexin V positive/7-PI negative cells by flow cytometry. Data are mean ± SEM, *n* = 5, *p < 0.05 versus EV −H_2_O_2_, ^#^*p* < 0.05 versus EV +H_2_O_2_ (one-way ANOVA). **(B)** Respiration was determined in isolated heart mitochondria of adult wild-type mice (“wt”) and p27-deficient littermates (“p27ko”), who had received drinking water without caffeine or water supplemented with 0.05% caffeine for 10 days. Respiration was measured as O_2_ consumption without the addition of substrates (“mito”) and after the successive addition of malate/glutamate (“M/G”), ADP, rotenone (“rot”), and succinate (“succ”) (left panel). The right panel shows a magnification of O_2_ consumption after the addition of M/G and ADP, respectively. Data are mean ± SEM, *n* = 5–8 per group, *p < 0.05 versus wt without caffeine (one-way ANOVA). **(C)** Adult p27-deficient animals and their wild-type littermates received drinking water or water supplemented with 0.05% caffeine for 10 days. RNAs were isolated from the hearts of those mice, and microarray analyses were conducted. Data are represented as a Venn diagram. The numbers in the circles indicate the number of transcripts regulated in the two genotypes (*n* = 3 animals per genotype and treatment, *p* < 0.05). Underlying data are provided in S1 Data. ADP, adenosine diphosphate; n.s., not significant; PI, propidium iodide.

It had been assumed that knockout of a cell cycle inhibitor like p27 could be beneficial in the experimental setup of myocardial infarction. This was based on the reasoning that myocardial infarction leads to loss of cells in the heart and that enhanced proliferation of cells in p27-deficient mice may result in smaller infarct size and reduced mortality. However, exactly the opposite was observed. Mice showed bigger infarct size, and the mortality was significantly increased [5,6]. Since functional mitochondria in the heart are required not only to provide energy for the pumping function but also to cope with externally or internally induced changes—e.g., during and after myocardial infarction—we hypothesized that a non-cell cycle–related function of p27, according to our data most likely in the mitochondria, could also be important for the heart. Therefore, we first analyzed the role of mitochondrial p27 in cell death induction in cardiomyocytes—a hallmark of cardiac pathologies [28]. We lentivirally expressed mitochondrially targeted p27 in cardiomyocytes and measured basal and oxidative stress-induced apoptosis. Mitochondrial p27 dramatically reduced basal apoptosis and completely blunted H_2_O_2_-induced cell death (Fig 5A). As functional mitochondria play a pivotal role in protection against heart disease and p27-deficient mice show increased mortality after myocardial infarction [6], we measured oxygen consumption in heart mitochondria isolated from adult p27-deficient mice and their wild-type littermates as a readout for mitochondrial function. Mitochondria isolated from p27-deficient mice displayed significantly reduced complex I respiration, which demonstrates that those animals have impaired mitochondrial functionality (Fig 5B). To further establish a causal link between caffeine and p27, p27-deficient animals were given 0.05% caffeine in drinking water for 10 days, a concentration for which we had previously shown to result in a serum concentration of approximately 30–50 μM and a time sufficient to completely restore the carotid endothelium after wire injury [8]. Strikingly, caffeine did not improve respiration in hearts of p27-deficient mice (Fig 5B), whereas respiration in wild-type littermates was increased by caffeine. Next, we wanted to determine whether a connection between caffeine and mitochondrial p27 also exists on the transcriptome level. Therefore, p27-deficient animals and their wild-type littermates were given 0.05% caffeine in drinking water for 10 days. After that, RNA was isolated from whole hearts, and microarray analyses were performed. As shown in the Venn diagram in Fig 5C, all but 3 of the 245 transcripts differentially expressed after caffeine administration in wild-type mice were p27-dependent, since only 3 were also regulated in p27-deficient animals. Interestingly, among the most highly enriched gene ontology (GO) categories for biological processes are GO terms describing pathways, which take place in the mitochondria (S1 Table). Strikingly, more than one-third of the transcripts in all other GO categories are translated into proteins localized in the mitochondria (S1 Table), demonstrating that the caffeine-induced, p27-dependent transcriptome changes affect to a large part the mitochondria.

### Mitochondrial p27 is required for proper cardiac myofibroblast differentiation

**Fig 6:**
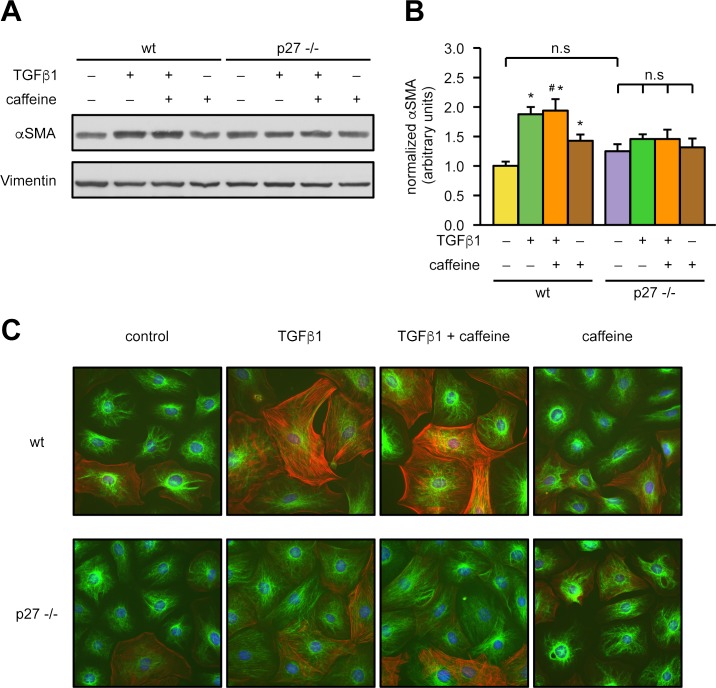
p27 is required for myofibroblast differentiation of cardiac fibroblasts. Cardiac fibroblasts were isolated from hearts of wild-type (“wt”) mice and p27-deficient (“p27 −/−”) littermates. Myofibroblast differentiation was induced by treatment with 2 ng/ml TGFβ1 for 48 hours in the presence or absence of 50 μM caffeine. Induction of αSMA was detected by immunoblot and immunostaining. **(A)** Representative immunoblots, Vimentin served as loading control. **(B)** Semiquantitative analysis of αSMA normalized to Vimentin. Data are mean ± SEM, *n* = 8: wt untreated, wt +TGFβ1, p27 −/− untreated, p27 −/− +TGFβ1; *n* = 5: all others, *p < 0.05 versus wt untreated, ^#^*p* < 0.05 versus wt +caffeine (one-way ANOVA). **(C)** Representative immunostainings: αSMA was stained in red and Vimentin in green, nuclei were counterstained with DAPI (blue), shown are the overlays of all fluorescence channels. Underlying data are provided in S1 Data. αSMA; α smooth muscle actin; DAPI, 4′,6-diamidino-2-phenylindole; n.s., not significant; TGFβ1, transforming growth factor β1.

Over the last several years, it has become evident that in several healing processes, including wound healing and the early phase after myocardial infarction, fibroblasts have to differentiate into myofibroblasts to fill the gaps caused by cell loss. Recent findings demonstrated that intact mitochondria are needed for differentiation of fibroblasts into myofibroblasts in response to factors like transforming growth factor β1 (TGFβ1) [9]. Thus, we isolated cardiac fibroblasts from p27-deficient mice and wild-type littermates and induced myofibroblast differentiation by TGFβ1 in the absence or presence of caffeine. TGFβ1 induced myofibroblast differentiation, measured by the up-regulation of α smooth muscle actin (αSMA), only in wild-type cardiac fibroblasts but not in p27-deficient cells (Fig 6). Moreover, caffeine treatment alone slightly but significantly increased αSMA levels, probably by improving mitochondrial function, again only in cells isolated from wild-type animals (Fig 6).

**Fig 7:**
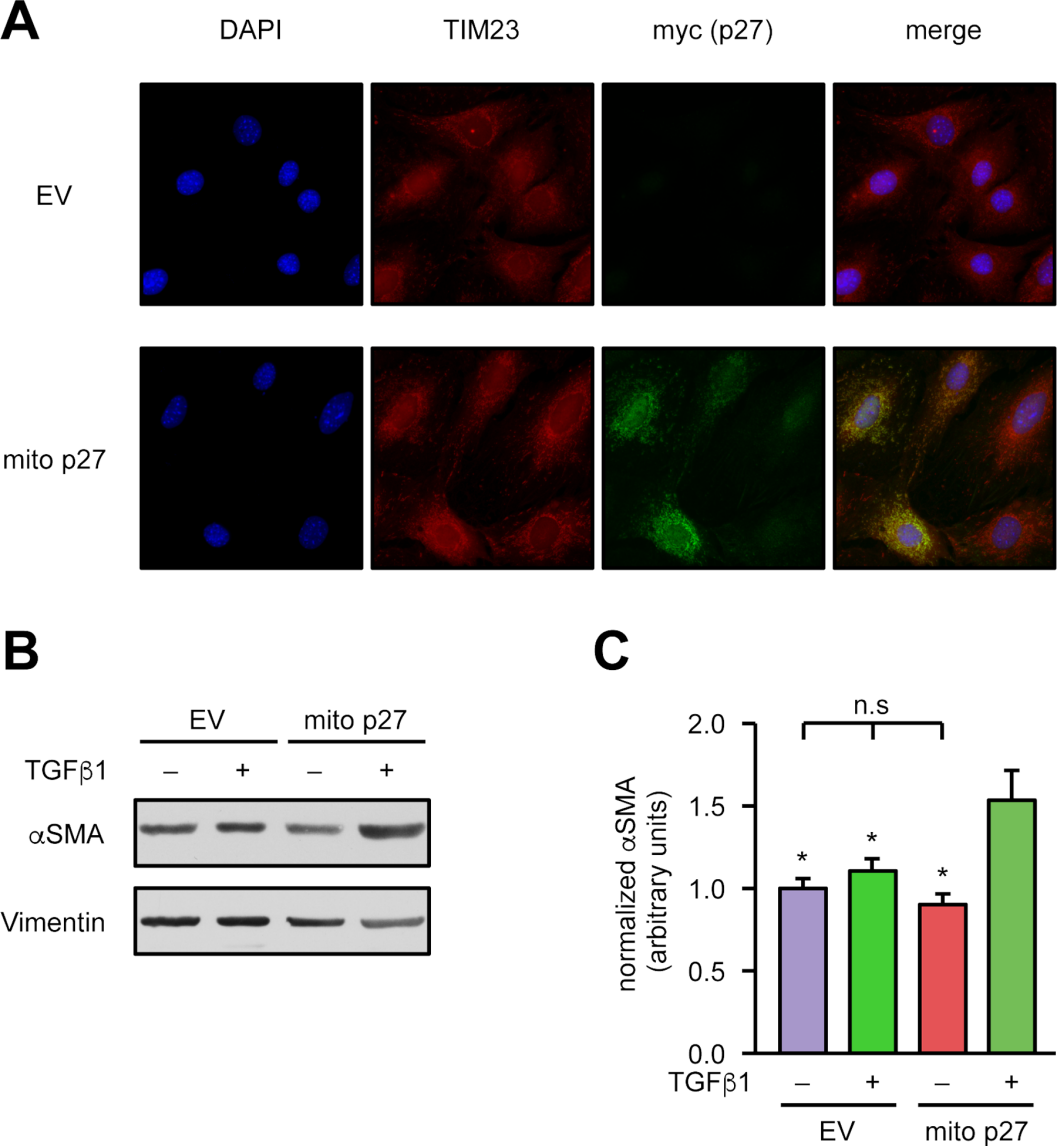
Mitochondrial p27 restores the impaired αSMA up-regulation in p27-deficient cardiac fibroblasts. Fibroblasts isolated from the hearts of p27-deficient mice were lentivirally transduced with an expression vector for mitochondrially targeted p27 (“mito p27”) or a corresponding empty vector (“EV”). **(A)** Representative immunostainings: nuclei were visualized with DAPI (blue), mitochondria by staining for TIM23 (red), and the mitochondrially targeted p27 by staining for the myc epitope (“myc (p27),” green). Merge shows an overlay of all fluorescence channels. **(B, C)** Myofibroblast differentiation was induced by treatment with 2 ng/ml TGFβ1 for 48 hours, and αSMA was detected by immunoblot. **(B)** Representative immunoblots, Vimentin served as loading control. **(C)** Semiquantitative analysis of αSMA normalized to Vimentin. Data are mean ± SEM, *n* = 5, *p < 0.05 versus mito p27 +TGFβ1 (one-way ANOVA). Underlying data are provided in S1 Data. αSMA; α smooth muscle actin; DAPI, 4′,6-diamidino-2-phenylindole; n.s., not significant; TGFβ1, transforming growth factor β1; TIM23, translocase of inner mitochondrial membrane 23.

To investigate whether mitochondrial p27 is sufficient to rescue the p27-deficient cells from the differentiation defect, we lentivirally expressed mitochondrially targeted p27 in p27-deficient cardiac fibroblasts. As demonstrated in Fig 7, reexpression of mitochondrial p27 restored the ability of p27-deficient cardiac fibroblasts to differentiate into myofibroblasts upon TGFβ1 treatment. Thus, we also established a causal link between mitochondrial p27 and the ability of fibroblasts to differentiate into myofibroblasts.

### Caffeine—in concert with mitochondrial p27—is protective in mouse models with mitochondrial dysfunction

**Fig 8:**
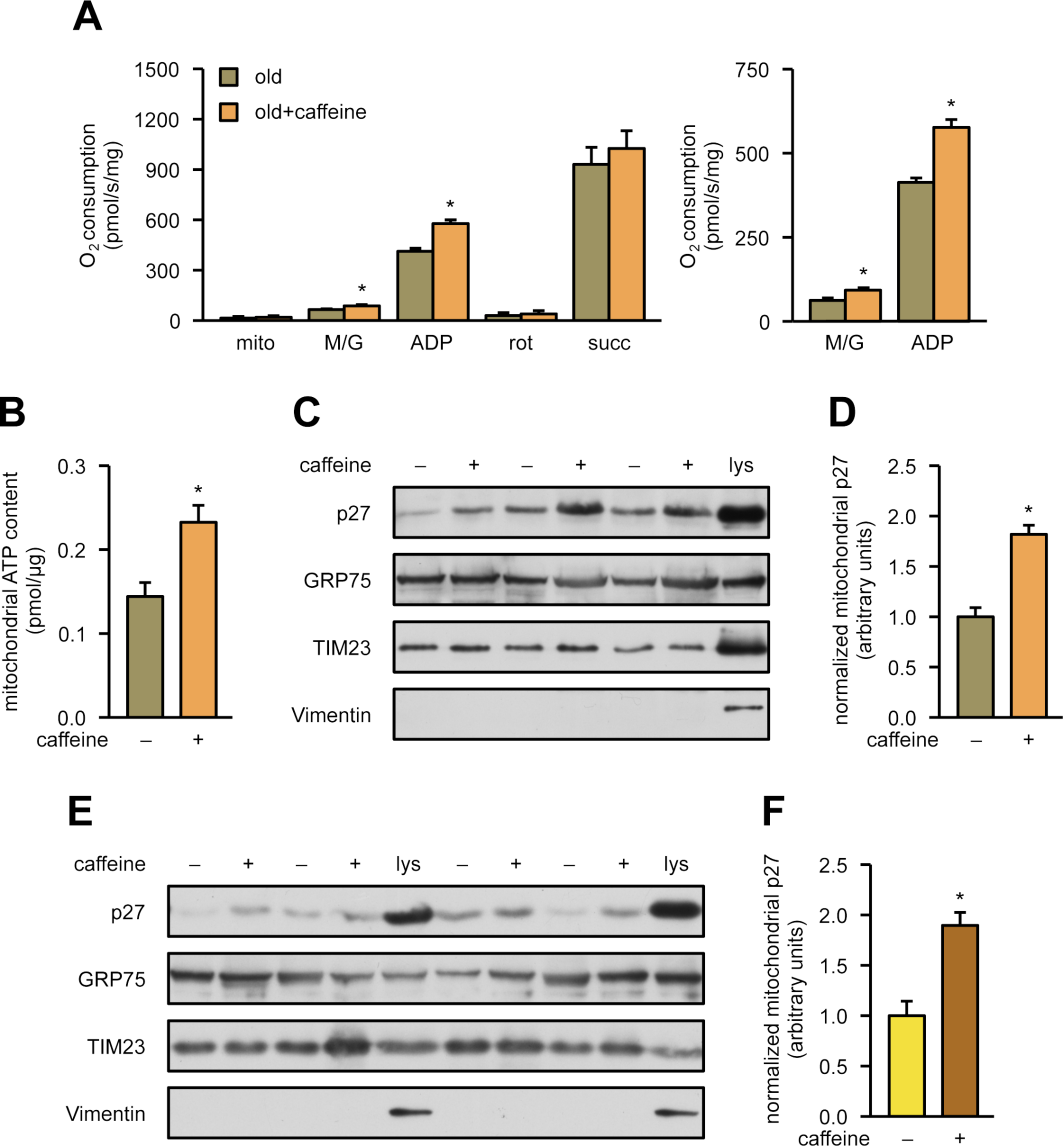
Caffeine enhances respiration, ATP content, and mitochondrial localization of p27 in old mouse hearts. **(A-D)** Twenty-two-month-old wild-type mice received drinking water (“old”) or water supplemented with 0.05% caffeine for 10 days (“old+caffeine”). **(A)** O_2_ consumption was measured in isolated heart mitochondria without the addition of substrates (“mito”) and after the successive addition of malate/glutamate (“M/G”), ADP, rotenone (“rot”), and succinate (“succ”) (left panel). The right panel shows a magnification of O_2_ consumption after the addition of malate/glutamate and ADP, respectively. Data are mean ± SEM, *n* = 6 per group, *p < 0.05 (one-way ANOVA). **(B)** Mitochondrial ATP content was measured with a luminometric assay. Data are mean, *n* = 5 per group, *p < 0.05 (one-way ANOVA). **(C)** Heart mitochondria were isolated, and p27 was detected by immunoblot; GRP75 and TIM23 served as loading controls. To control for purity of the mitochondria, a total heart lysate (“lys”) was used in parallel, and Vimentin was detected. Shown is a representative immunoblot. **(D)** Semiquantitative analysis of mitochondrial p27; data are mean ± SEM, *n* = 7 per group, *p < 0.05 (one-way ANOVA). **(E, F)** Six-month-old wild-type mice received drinking water or water supplemented with 0.05% caffeine for 10 days. **(E)** Heart mitochondria were isolated, and p27 was detected by immunoblot; GRP75 and TIM23 served as loading controls. To control for purity of the mitochondria, a total heart lysate (“lys”) was used in parallel, and Vimentin was detected. Shown is a representative immunoblot. **(F)** Semiquantitative analysis of mitochondrial p27; data are mean ± SEM, *n* = 5 per group, *p < 0.05 (one-way ANOVA). Underlying data are provided in S1 Data. ADP, adenosine diphosphate; ATP, adenosine triphosphate; GRP75, 75 KDa glucose-regulated protein; TIM23, translocase of inner mitochondrial membrane 23.

One hallmark of the murine and human aging process is reduced mitochondrial respiratory capacity. Therefore, we wanted to determine whether a 10-day treatment with caffeine in 22-month-old mice could enhance respiration. Indeed, caffeine increased respiration (Fig 8A). Moreover, the mitochondrial ATP content was increased to roughly the same extent as the mitochondrial oxygen consumption of complex I (Fig 8B). Interestingly, mitochondrial respiration in hearts of adult p27-deficient mice was similar as in 22-month-old wild-type animals (S6 Fig), suggesting that loss of mitochondrial p27 impairs the heart as strongly as aging. This is in accordance with increased infarct size and early mortality after myocardial infarction in p27-deficient mice [6]. Furthermore, 10 days of caffeine treatment in old animals was sufficient to raise the mitochondrial respiration to the levels observed in 6-month-old mice (S6 Fig). In addition, the analysis of cardiac mitochondria from old mice showed a roughly 2-fold increase in mitochondrial p27 content after 10 days of caffeine (Figs 8C and 8D and S7), demonstrating that caffeine-induced improved respiration is paralleled by an increase in mitochondrial p27. The amount of mitochondrial p27 in heart mitochondria of old mice after caffeine consumption was comparable to mitochondrial p27 in heart mitochondria of 6-month-old mice (S6 Fig). Thus, treatment of old mice with caffeine for 10 days markedly improved mitochondrial p27 and thus respiration in the heart. In addition, we also treated adult 6-month-old littermates with caffeine for 10 days and analyzed mitochondrial p27 by immunoblot. Similar to old mice, caffeine also increased mitochondrial p27 in adult 6-month-old mice when compared to their wild-type littermates (Fig 8E and 8F).

**Fig 9:**
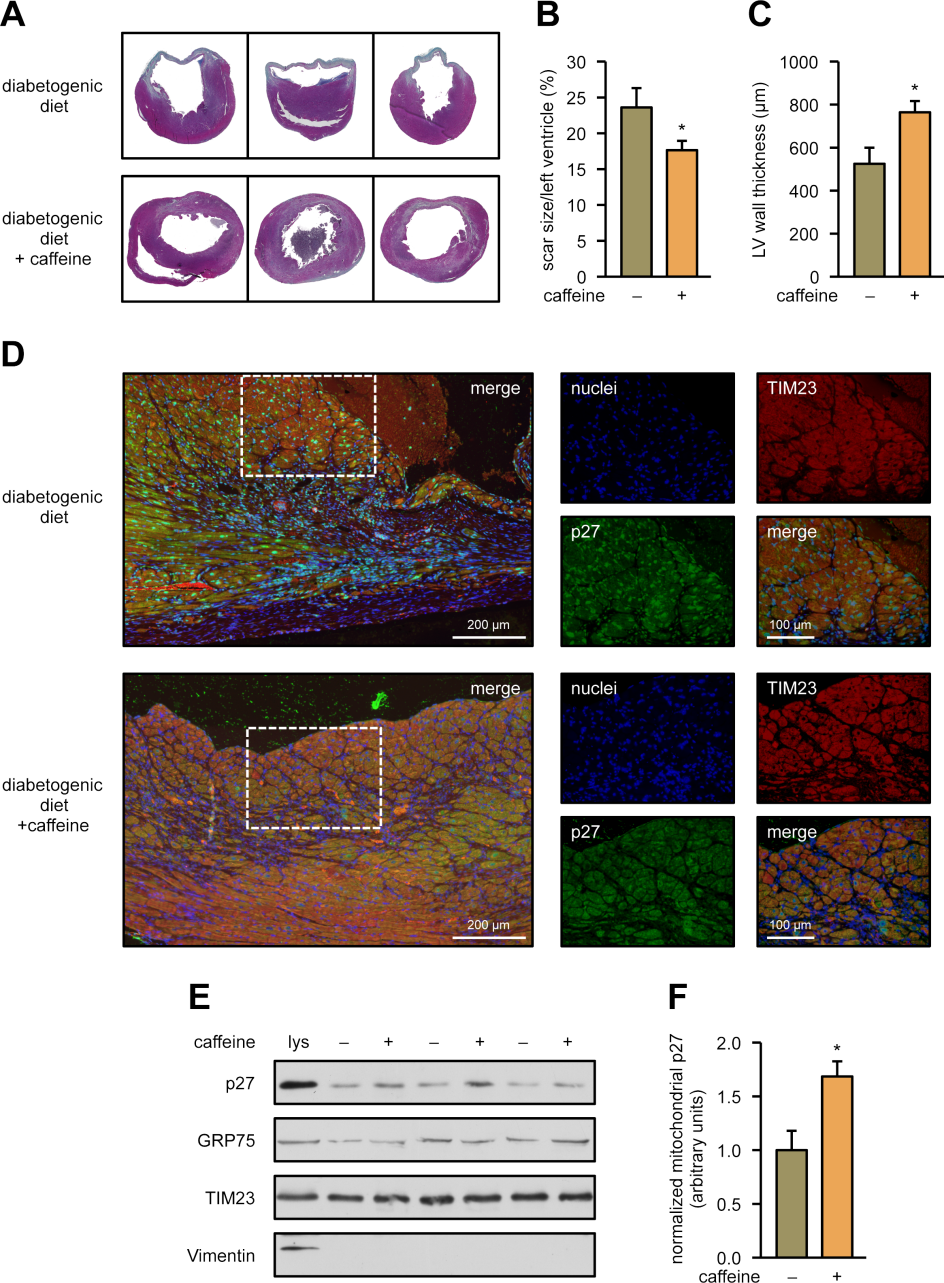
Caffeine improves outcomes after myocardial infarction in prediabetic mice and induces mitochondrial translocation of p27. Two-month-old wild-type mice were fed a diabetogenic diet for 11 weeks. For the last 10 days, one group of animals received drinking water supplemented with 0.05% caffeine. Afterward, myocardial infarction was induced by ligation of the left anterior descending coronary artery for 60 minutes followed by reperfusion. Twenty-one days after infarction, hearts were excised, sectioned, and the sections stained. **(A)** Representative Gomori stainings of sections of 3 different hearts for each dietary regimen. **(B)** Infarct size per left ventricle and **(C)** minimum left ventricular (“LV”) wall thickness in the infarcted myocardium. Data are mean ± SEM, *n* = 8: diabetogenic diet, *n* = 10: diabetogenic diet +caffeine, *p < 0.05 (one-way ANOVA). **(D)** Representative immunostainings of border zone sections for each dietary regimen. TIM23 is stained in red, p27 in green, nuclei were counterstained with DAPI (blue), merge shows an overlay of all fluorescence channels. The dotted rectangles indicate the sections shown in higher magnifications. **(E)** Heart mitochondria were isolated, and p27 was detected by immunoblot; GRP75 and TIM23 served as loading controls. To control for purity of the mitochondria, a total heart lysate (“lys”) was used in parallel, and Vimentin was detected. Shown is a representative immunoblot. **(F)** Semiquantitative analysis of mitochondrial p27; data are mean ± SEM, *n* = 5, *p < 0.05 (one-way ANOVA). Underlying data are provided in S1 Data. DAPI, 4′,6-diamidino-2-phenylindole; GRP75, 75 KDa glucose-regulated protein; TIM23, translocase of inner mitochondrial membrane 23.

Not only aging but also obesity and type 2 diabetes have been demonstrated to be associated with mitochondrial dysfunction [29,30]. Therefore, we used a second animal model in which we fed 2-month-old mice a diabetogenic diet (S2 Table) for a total of 9.5 weeks, leading to obesity and a prediabetic state. After that, mice were separated into 2 groups, one of which received caffeine in the drinking water for 10 days. Then, ischemia reperfusion injury was set, and animals were analyzed 3 weeks later. We first measured the scar size of the left ventricle and the minimum left ventricular wall thickness in both groups. Ten days of caffeine treatment significantly reduced scar size and improved wall thickness (Fig 9A, 9B and 9C). Next, we investigated whether caffeine induces translocation of p27 into the mitochondria in this mouse model analogous to our observations in cells and in healthy adult as well as in old mice. Therefore, coimmunostainings of heart slices for p27 and the inner mitochondrial membrane protein TIM23 were performed in the border zone of the infarcted area. Indeed, colocalization of p27 and TIM23 was increased in the animals that had received caffeine, whereas p27 was mostly nuclear in the hearts of the mice on the diabetogenic diet without caffeine supplementation (Fig 9D). To further support the results obtained in tissue slices of the heart, we isolated mitochondria from hearts of mice fed a diabetogenic diet for 11 weeks, with the last 10 days on drinking water or water supplemented with caffeine. In accordance with our coimmunostainings in Fig 9D, p27 was significantly increased in the mitochondria of mice that had received caffeine in their drinking water (Figs 9E, 9F and S7).

These data demonstrate that caffeine treatment in obese mice can reduce myocardial infarction injury and, in parallel, increase the levels of mitochondrial p27.

## Discussion

Here, we demonstrate that p27 is localized in the mitochondria. Serine 10 and threonine 187 within p27 are required for import into the mitochondria and functional improvements induced by mitochondrial p27. Moreover, mitochondrial p27 is sufficient to improve cellular processes, which depend on functional mitochondria, in different cells of the cardiovascular system. Moreover, it is suggestive to assume that the translocation of p27 into mitochondria might be critically involved in the improved outcomes after myocardial infarction upon caffeine administration. In summary, we present an increase in mitochondrial p27 as a new mode of action for how measurable caffeine concentrations in humans improve the functionality of the cardiovascular system or can even be protective in states associated with increased risk for cardiovascular diseases.

p27 was initially discovered as a nuclear-localized cell cycle inhibitory protein [1]. Previous data demonstrating that p27 can be exported to the cytoplasm [2,3] were considered as a mechanism to inactivate the cell cycle inhibitory effects of p27 in the nucleus and to allow human cancer cells to escape cell cycle arrest. However, McAllister and colleagues demonstrated that nonnuclear p27 is required for migration of fibroblasts, since p27-deficient mouse embryonic fibroblasts failed to migrate, while reconstitution with p27 rescued the motility defect [4]. Here, we show that only mitochondrial p27—but not nuclear p27—rescues the loss of migratory capacity induced by knockdown of endogenous p27, revealing a causal, direct link between mitochondrial localization of p27 and endothelial cell migration. Moreover, serine 10 and threonine 187 are required for import into mitochondria and the promigratory action of mitochondrial p27.

Interestingly, p27 is not the only protein initially described as a cell cycle inhibitor that was subsequently shown to elicit cytoplasmic and mitochondrial functions. In fact, prohibitin and prohibitin-2 were originally characterized as tumor suppressor proteins with antiproliferative activity when present in the nucleus [31–33]. However, when localized in the mitochondria, prohibitins act as mitochondrial membrane–bound chaperones for the stabilization of mitochondrial proteins [34], and interaction of prohibitin with subunits of complex I of the respiratory chain increases mitochondrial activity [35]. Similarly, we show here that p27 is localized within the mitochondria, where it improves mitochondrial functions. Interestingly, prohibitin has also been shown to be required for cell migration [36]. Thus, it is tempting to speculate that mitochondrial p27 exerts chaperone and/or assembly functions by interacting with mitochondrial proteins such as prohibitins, in analogy to nuclear p27, which is required for cyclin D/CDK complex assembly [37]. It is important to note that mitochondrial import of the nonphosphorylatable p27 S10A/T187A mutant is markedly impaired. Thus, serine 10 and threonine 187 are required not only for p27 functions within the mitochondria but also for its import into these organelles. Similar to the p27 S10A/T187A mutant, the mutant that lacks larger regions of the N- and C-termini also showed an impaired impact on migratory capacity compared to intact p27, even when exclusively localized in the mitochondria. These results confirm the importance of the N- and C-termini of mitochondrial p27 and therein serine 10 and threonine 187 for improving migration of endothelial cells.

Importantly, preserved endothelial cell function accounts for up to 40% of insulin-mediated glucose metabolism in humans [38]. Thus, caffeine-mediated stimulation of the functional capacity of the endothelium may indeed provide a direct mechanistic link for the inverse relationship between habitual coffee consumption and the risk for developing type 2 diabetes mellitus [11]. Moreover, our results with the diabetogenic diet in mice demonstrate that caffeine reduces infarct size in obese, prediabetic mice. Since obesity and type 2 diabetes mellitus increase the risk for myocardial infarction [39,40], coffee consumption—in addition to adequate medication, body weight lowering, and moderate exercise—could help to reduce this risk. Two large cohort studies revealed an association between coffee drinking and reduced mortality. In a prospective study of the National Institutes of Health, coffee drinking was inversely associated with subsequent mortality among 229,119 men and 173,141 women for deaths due to heart disease, respiratory disease, stroke, injuries and accidents, diabetes, and infections [12]. Similar results were obtained in a study with 521,330 participants in 10 European countries [13].

With respect to aging and thus to the elderly population, our data demonstrate that the mitochondrial capacity of the old heart is improved by caffeine to that of the adult heart. Since improving cardiovascular functionality in the elderly population is of major importance for extending health span, coffee consumption or caffeine per se could be considered as an additional protective dietary factor for the elderly population. Indeed, epidemiological analyses provided evidence that habitual intake of caffeinated beverages reduces the risk of heart disease mortality among elderly [14,15]. Moreover, since the caffeine effects are linked to increased mitochondrial p27 and thus improved mitochondrial function, enhancing mitochondrial p27 could serve as a potential therapeutic strategy not only in cardiovascular diseases but also in improving health span.

## Materials and methods

### Ethics statement

The study does not involve human participants and/or tissue. All experimental protocols for animal studies were approved by the Animal Ethics Committee of the LANUV, Duesseldorf (Az.: 84–02.05.50.15.023, Az.: 84–02.04.2016.A204, Az.: 84–02.04.2015.A322). The anesthetics used are detailed in the sections “Preparation of mouse heart mitochondria” and “Myocardial ischemia and reperfusion”.

### Experimental animals

p27-deficient mice (B6.129S4-*Cdkn1btm1Mlf*/J) [41] were originally obtained from V. Andres (Madrid, Spain) and backcrossed onto C57BL/6NTac (Taconic) for more than 10 generations. Only heterozygous p27-deficient animals were used as breeders, and the offspring was genotyped with a multiplex PCR using DNA prepared from tail clips with the DirectPCR Lysis Reagent (Mouse Tail; Viagen Biotech). The primers used were p27ko for1 (5′-AGTTGTGCCTTGTATGCTGGT-3′), p27ko rev1 (5′-ACAACAAGCTGGAACCCTGT-3′), and mPGKpA for1 (5′-ATTAAGGGCCAGCTCATTCC-3′). Amplifications were performed for 10 cycles with an annealing temperature starting at 65°C and a decrease of 1°C per cycle, followed by 30 cycles with a constant annealing temperature of 56°C; the extension time in all cycles was 30 seconds. Amplification products were resolved on 1.5% agarose gels, the wild-type allele yields a product of 553 bp, the null allele a product of 325 bp. For all experiments, in which no p27-deficient littermates were required, C57BL/6 animals were purchased from Janvier.

### Isolation and cultivation of cardiac fibroblasts and induction of myofibroblast differentiation

Mice were sacrificed by cervical dislocation, the hearts were excised, and all fat and large vessels were removed with a scalpel. Hearts were placed in a culture dish with room-temperature PBS (Thermo Fisher Scientific) supplemented with 1% penicillin/streptomycin (Thermo Fisher Scientific) and 2 mM CaCl_2_ (PBS^(++)^), and the blood was squeezed out with tweezers. After transfer to a new culture dish with PBS^(++)^, hearts were chopped into small pieces. The pieces were distributed into two 2-ml Eppendorf tubes, each containing 1 ml of a freshly prepared, ice-cold collagenase solution (1 U/ml Collagenase NB 8 Broad Range [Serva] in PBS^(++)^, filter sterilized), and incubated for 15 minutes at 37°C with gentle mixing every 5 minutes. The cell-containing supernatants were transferred to 2-ml Eppendorf tubes containing DMEM GlutaMAX (Thermo Fisher Scientific) supplemented with 20% fetal bovine serum (Thermo Fisher Scientific) and 1% penicillin/streptomycin to stop the collagenase reaction. After centrifugation for 5 minutes at 400 xg at 4°C, the pelleted cells were resuspended in 1 ml DMEM GlutaMAX/20% fetal bovine serum/1% penicillin/streptomycin and placed on ice. In parallel, the remainder of the heart pieces was digested again with collagenase under identical conditions. The collagenase digestions were repeated until no more pieces were visible. Finally, all cells were pooled, plated onto a 10-cm culture dish, and placed in a humidified tissue culture incubator at 37°C in an atmosphere containing 5% CO_2_. After 2 hours, all nonadherent cells were carefully aspirated off. Attached cells were washed twice with DMEM GlutaMAX/10% fetal bovine serum/1% penicillin/streptomycin and from then on grown in this medium.

### Cell culture

All cells were cultivated in a humidified tissue culture incubator at 37°C in an atmosphere containing 5% CO_2_. Primary human endothelial cells were obtained from Lonza and cultured in endothelial basal medium supplemented with 1 μg/ml hydrocortisone, 12 μg/ml bovine brain extract, 50 μg/ml gentamicin, 50 ng/ml amphotericin B, 10 ng/ml epidermal growth factor (Lonza), and 10% fetal bovine serum until the third passage. After detachment with trypsin, cells were grown for at least 18 hours before transfection or treatment. All experiments were performed in the presence of complete medium including 10% fetal bovine serum.

The murine cardiac muscle cell line HL-1 [42] was a gift from W. C. Claycomb and was cultivated in Claycomb medium (Sigma Aldrich) supplemented with 1% penicillin/streptomycin, 100 μM norepinepherine (Sigma Aldrich), 2 mM L-glutamine (Sigma Aldrich), and 10% fetal bovine serum for as many passages as the cells showed contractile activity in the culture dish.

The human embryonic kidney cell line HEK293FT was obtained from Invitrogen and cultured in DMEM GlutaMAX supplemented with 10% heat-inactivated fetal bovine serum, 1% penicillin/streptomycin, 0.5 mg/ml geneticin (Thermo Fisher Scientific) as selective antibiotic, and 1% nonessential amino acids (Thermo Fisher Scientific).

Cardiac fibroblasts were cultivated in DMEM GlutaMAX supplemented with 10% fetal bovine serum and 1% penicillin/streptomycin (Thermo Fisher Scientific). For the induction of myofibroblast differentiation, the cells were grown for 24 hours in DMEM GlutaMAX/1% fetal bovine serum/1% penicillin/streptomycin before recombinant human TGFβ1 (2 ng/ml; Peprotech) was added for another 48 hours.

Cell lines and primary murine cardiac fibroblasts were routinely tested to be free of mycoplasmas using a PCR-based approach, which detects the most common species of mycoplasmas and includes appropriate internal and positive controls [43].

### Transient transfections

Endothelial cells were transfected on 6-cm culture dishes with 3 μg plasmid DNA and 25 μl Superfect (Qiagen) as described previously, with a transfection efficiency of 40% [44]. Endogenous p27 was down-regulated by transfection with 2 different siRNAs (p27 siRNA-1 duplex sense strand: 5′-GCGCAAGUGGAAUUUCGAU-3′; p27 siRNA-2 duplex sense strand: 5′-GAGCCAACAGAACAGAAGA-3′) using JetSi reagent (Eurogentec) according to the manufacturer’s instructions. Expression of nuclear- or mitochondrially targeted p27 after knockdown of the endogenous protein was achieved by transfection with Superfect (Qiagen) 18 hours later, using Superfect as described above.

### Measurements of cell viability with 3-(4,5-dimethylthiazol-2-yl)-2,5-diphenyltetrazolium (MTT)

Cells were incubated with 0.25 mg/ml MTT in medium for 4 hours. After removing the medium, cells were washed with PBS, and formazan crystals were dissolved with dimethyl sulfoxide (DMSO). The resulting supernatant was measured in a TECAN plate reader at an absorbance of 550 nm. Absorbance of DMSO at 550 nm was subtracted as background.

### Scratch wound assay of endothelial cells

For detection of cell migration, wounds were created by scraping confluent cell monolayers with a sterile disposable rubber policeman [45]. Therefore, endothelial cells were grown on 6-cm dishes, which were previously labeled with a trace line. After injury, nonattached cells were removed by gently washing with culture medium. In cases in which migration of transfected cells was analyzed, the wound was set 5 hours after transfection. For caffeine treatments, caffeine was added after the wound was set. Endothelial cell migration from the edge of the injured monolayer was quantified by staining the cells with 20 ng/ml 4′,6-diamidino-2-phenylindole (DAPI; Carl Roth) in PBS after the cells had been fixed with 4% paraformaldehyde for 15 minutes at room temperature. Microscopic pictures were taken using a Zeiss Axiovert 100, and the cells, which had invaded the wound from the trace line, were automatically counted using the particle analysis feature of ImageJ 1.42q [46] after watershed separation of overlapping nuclei.

### Cloning of p27 expression vectors

The human p27 coding sequence (NM_004064) without the translation termination codon was amplified from endothelial cell cDNA with primers containing Sal I and Not I restriction sites and inserted into pCMV/myc/nuc and pCMV/myc/mito (Invitrogen) opened with these enzymes to generate expression vectors for nuclear and mitochondrially targeted p27, respectively. An analogous expression vector for nontargeted p27 was created by inserting the p27 coding sequence into pCMV/myc/cyto (Invitrogen). Deletion mutants were created by amplifying subregions of the p27 coding sequence with appropriate primers and insertion into the same vector backbones. Point mutations were introduced by site-directed mutagenesis using the QuikChange Multi Site-Directed Mutagenesis Kit (Agilent Technologies). The starting plasmids were used as empty vectors in the respective transfection experiments.

The lentiviral transfer vector for the expression vector of mitochondrially targeted p27 was created by inserting a DNA fragment containing the CMV promoter and the p27 coding sequence with the N-terminal mitochondrial targeting sequence from the expression vector for mitochondrially targeted p27 into pLKO.1-puro (Sigma Aldrich), which also served as an empty vector for the respective transductions.

The identity of all plasmids was verified by restriction digestion and DNA sequencing. Plasmid DNAs for transfections were purified with the HiSpeed Plasmid Maxi kit (Qiagen) according to the manufacturer's specifications. Concentrations were measured spectrophotometrically using a Nanodrop, and the identity and purity of each preparation was reconfirmed by restriction digestion.

### Lentiviral production and transduction

VSV-G pseudotyped lentiviral transduction particles were generated as previously described [47]. Briefly, HEK293FT cells were cotransfected with a transfer vector and expression vectors for the VSV-G envelope protein and lentiviral Gag/Pol, using the Calcium Phosphate Transfection Kit (Invitrogen) according to the manufacturer's instructions. Virus-containing culture supernatants were collected over several days, filtered through a 0.45 μm PVDF membrane, and concentrated by ultrafiltration using Vivacell 100 ultrafiltration units with a PES membrane and a molecular weight cutoff of 100.000 (Sartorius). Concentrated virus particles were dispensed in aliquots and stored at −80°C. Viral titers were determined with the QuickTiter Lentivirus Titer Kit (Lentivirus-Associated HIV p24; Cell Biolabs). HL-1 cells or murine cardiac fibroblasts were transduced with a multiplicity of infection of approximately 20. The day after transduction, the cells were washed 3 times, the medium was replaced, and the H_2_O_2_ treatment was started.

### Total cell lysis

Cells were scraped off the plates and centrifuged for 10 minutes at 800 xg at 4°C. After washing with PBS, cells were resuspended in RIPA-buffer (50 mM Tris/HCl pH 8, 1% IGEPAL CA-630, 150 mM NaCl, 0.1% SDS, 0.5% desoxycholate) supplemented with protease inhibitor cocktail and phosphatase inhibitor cocktail (both Bimake) and lysed for 30 minutes at 4°C. Lysates were centrifuged at 18.000 xg, and supernatants were transferred to fresh, precooled Eppendorf tubes.

### Fractionation of cells

Cells were scraped off the plates and centrifuged for 10 minutes at 800 xg at 4°C. After washing with PBS, cells were resuspended in mitochondrial isolation buffer (20 mM HEPES, pH 7.4, 10 mM KCl, 5 mM MgCl_2_, 1 mM EDTA, 1 mM EGTA, 250 mM sucrose), incubated for 3 minutes on ice, and then disrupted using a Dounce homogenizer. Cellular debris was removed by centrifugation for 10 minutes at 3.000 xg at 4°C. The resulting supernatant was transferred to a new tube and centrifuged again for 15 minutes at 10.000 xg at 4°C. The resulting pellet was washed at least 3 times with mitochondrial isolation buffer. Finally, the pellet was resuspended in mitochondrial isolation buffer and used for further analyses. The resulting supernatant was collected as a nonmitochondrial fraction.

### Proteinase K digestion of mitochondria

Proteinase K digestion of mitochondria was performed essentially as previously described by us [53]. Briefly, to determine where in the mitochondria a protein is localized, 300 μg of mitochondria were distributed in 4 equal aliquots. Mitochondria were pelleted for 5 minutes at 10.000 xg at 4°C and incubated at 4°C on a shaker in 40 μl of 3 different buffers for 20 minutes. Buffer 1 (isotonic buffer): 250 mM sucrose, 1 mM EGTA, 10 mM HEPES, pH 7; Buffer 2 (hypotonic buffer): 1 mM EGTA, 10 mM HEPES, pH 7, 25 μg/ml proteinase K; Buffer 3 (hypotonic buffer with detergent): 1 mM EGTA, 10 mM HEPES, pH7, 1% Triton-X100, 25 μg/ml proteinase K. After 20 minutes, digestion was stopped by adding phenylmethylsulfonyl fluoride to a final concentration of 2 mM, and incubation continued for a further 5 minutes with shaking. Aliquot 3 was boiled for 5 minutes in Laemmli-buffer. Aliquot 1 and 2 were washed once with Buffer 1 and resuspended in 40 μl RIPA-buffer (50 mM Tris/HCl pH 8, 1% IGEPAL CA-630, 150 mM NaCl, 0.1% SDS, 0.5% desoxycholate) and boiled for 5 minutes in Laemmli-buffer.

### Sodium dodecyl sulfate polyacrylamide gel electrophoresis (SDS-PAGE) and immunoblotting

Electrophoretic separation of proteins with SDS-PAGE and blotting onto polyvinylidene difluoride membranes were performed according to standard methods. Detection of the different proteins was performed with antibodies directed against p27 (clone D37H1, Cell Signaling Technology, 1:300), phospho-p27 (S10; clone EP233(2)Y, Abcam, 1:300), phospho-p27 (T187; polyclonal, ab75908, Abcam, 1:300), TIM23 (clone 32, BD Biosciences, 1:2,000), TOM40 (polyclonal sc11414 and monoclonal, sc365467, Santa Cruz Biotechnology, 1:400), Trx-1 (clone 3A1, Abcam, 1:1,000), GRP75 (clone D13H4, Cell Signaling Technology, 1:500), γ-Actin (clone 2–2.1.14.17, Sigma Aldrich,1:5,000), α-Tubulin (clone DM1A, Sigma Aldrich, 1:50,000), myc-tag (rabbit clone 71D10 or mouse clone 9B11, Cell Signaling Technology, 1:500), Vimentin (clone EPR3776, Abcam, 1:12,000), αSMA (polyclonal, ab5694, Abcam, 1:6,000), PDE5A (polyclonal, #2395, Cell Signaling Technology, 1:10,00), phospho-PDE5A (S92 in mouse, S102 in human; polyclonal, GTX36930, Genetex, 1:250), PDE4A (polyclonal, ab200383, Abcam, 1:500), and phospho-PDE4A (serine 686/688; polyclonal, NB300-635, Novus Biological, 1:1,000). After protein transfer, membranes were incubated with primary antibodies overnight at 4°C before they were washed and incubated with secondary antibodies (anti-mouse IgG, HRP-linked whole Ab from sheep, NA931V, GE Healthcare Life Sciences, anti-rabbit IgG, HRP-linked whole Ab from donkey, NA934V, GE Healthcare Life Sciences) according to standard procedures. Detection was performed by enhanced chemiluminescence using the ECL reagent (GE Healthcare) and standard X-ray films. Semiquantitative analyses were performed on scanned X-ray films using ImageJ 1.42q [46].

### ATP measurements

ATP levels in total cell lysates and mitochondria preparations were determined with the luminescence-based ATP Kit SL (BioThema). ATP concentrations were calculated according to the manufacturer's recommendations.

### Mitochondrial membrane potential

JC1 dye exhibits potential-dependent accumulation in mitochondria, indicative by a fluorescence emission shift from green (approximately 529 nm) to red (approximately 590 nm). Consequently, mitochondrial depolarization is indicated by a decrease in the red/green fluorescence intensity ratio. Therefore, cells were incubated with JC1 at a final concentration of 0.5 μM for 30 minutes. Cells were washed twice with PBS, and fluorescence intensities were determined using a FACSCalibur (Becton Dickinson). Mean red JC1 fluorescence was calculated.

### Immunostaining of cells

For the detection of nuclear- and mitochondrially targeted p27, cells were fixed in 4% paraformaldehyde and permeabilized using 0.3% Triton-X 100/3% bovine serum albumin in PBS. For coimmunostaining, cells were first incubated with a mouse antibody against myc-tag (clone 9E10, Santa Cruz Biotechnology, 1:50) at 4°C overnight, and a Rhodamine Red-X-conjugated Fab fragment anti-mouse was used as secondary antibody (Jackson ImmunoResearch, 1:300, 1 hour, room temperature). Afterward, cells were incubated with a rabbit anti-TOM40 antibody (polyclonal, sc11414, Santa Cruz Biotechnology, 1:50) at room temperature overnight and an Alexa 488 anti-rabbit secondary antibody (Invitrogen, 1:200, 1 hour, room temperature). Nuclei were counterstained with DAPI.

For the localization studies of mitochondrially targeted p27 deletion mutants, endothelial cells were stained for mitochondria using Mito Tracker Red CMXRos (Thermo Fisher Scientific, 1:50,000, 30 minutes, room temperature). Subsequently, cells were washed with PBS and fixed for 15 minutes with 4% paraformaldehyde. For permeabilization, 0.3% Triton X-100 and 3% bovine serum albumin in PBS were used for 15 minutes. Afterward, cells were incubated with an FITC-coupled anti-myc-tag antibody (clone 9E10, Santa Cruz Biotechnology, 1:50) at 4°C overnight. Nuclei were visualized with 20 ng/ml DAPI in PBS. Cells were washed with PBS and mounted with ProLong Gold antifade mounting medium (Invitrogen).

A direct immunostaining of αSMA and Vimentin was performed in mouse cardiac fibroblasts. Cells were fixed and permeabilized as described above. An Alexa-Fluor 594 conjugated antibody against αSMA (clone 1A4, Abcam, 1:100) and an Alexa-Fluor 488 conjugated anti-Vimentin antibody (clone, D21H3, Cell Signaling Technology, 1:100) were incubated at 4°C overnight. Afterward, nuclei were stained with DAPI, and cells were mounted as above. All primary antibodies were diluted in PBS containing 1% bovine serum albumin.

Fluorescence images were taken with a Zeiss AXIOVERT 200 M or a Zeiss Axio Imager M2.

### Apoptosis measurement

Detection of apoptosis was performed by flow cytometry using annexin V–APC binding and 7-amino-actinomycin (7-AAD) staining as described previously [48]. Only annexin V positive/ 7-AAD negative cells were counted truly apoptotic.

### RNA isolation and microarrays

RNA was isolated from mouse hearts using Trizol according to the manufacturer’s instruction (Invitrogen) and subjected to a second purification step using RNeasy columns (Qiagen). RNA integrity was checked on an Agilent 2100 Bioanalyzer, and concentrations were determined by photometric Nanodrop measurement. All samples in this study showed common high-quality RNA Integrity Numbers (RIN 9.7–10).

To study the differences in gene expression between wild-type mice and their p27-deficient littermates in response to caffeine, we used oligonucleotide-based microarrays. The *Mus musculus* AROS Oligo Set V4.0 was obtained from Operon. Oligonucleotides (70 mers) were dissolved in amino spotting buffer to a concentration of 20 μM (Genetix) and spotted onto UltraGap slides (Corning). After the printing process, the oligonucleotides were UV cross-linked (630 mJ/cm2) to the slide surface (NCBI Gene Expression Omnibus Platform GPL5403).

Labeled cRNA probes were synthesized from 500 ng of total RNA using the Quick Amp Labeling Kit (one-color; Agilent Technologies) according to the manufacturer’s protocol. Prior to hybridization, the slides were incubated in a prewarmed BSA blocking solution containing 5x SSC, 0.1% SDS, and 0.1 mg/ml BSA at 42°C for 45 to 60 minutes. Subsequently, slides were rinsed twice in 0.1x SSC for 5 minutes and for 30 seconds in double-distilled water, both at room temperature. The slides were then dried in a nitrogen flow. Cy3-labeled cRNA samples (2.5 μg) were dissolved in hybridization buffer (final concentration 50% formamide, 5x SSC, 0.1% SDS). Hybridization was carried out in a humid chamber at 42°C for 16 hours. After the hybridization step, unbound cRNA and hybridization buffer were removed by several washing steps (2 times for 10 minutes 2x SSC, 0.1% SDS; 5 times for 1 minute 0.1x SSC; and 10 seconds 0.01x SSC).

Fluorescence signals were visualized by a GenePix 4000B laser scanner (Axon). GenePix Pro software (v. 6.0) was used to calculate fluorescence intensities. Data analyses on microarray probe signal intensities were conducted with GeneSpring GX software (v. 11.0.2; Agilent Technologies). Probe signal intensities were quantile normalized across all samples to reduce interarray variability. Input data preprocessing was concluded by baseline transformation to the median of all samples. To further improve signal-to-noise ratio, a given probe had to be expressed above background (i.e., fluorescence signal of the probe was detected within the 20th and 100th percentiles of the raw signal distribution of a given array) in all 3 replicates in at least 1 of 2 or both conditions to be subsequently analyzed in pairwise comparisons. Differential gene expression was statistically determined by unpaired *t* tests. The significance threshold was set to *p* < 0.05.

GO analyses were performed using DAVID [49,50]. GO category enrichment was statistically evaluated by modified Fisher Exact testing in DAVID (EASE scoring). Additionally, fold enrichment was determined as the ratio of 2 proportions: (1) number of genes associated with a defined biological process in the experimental data set/total number of differentially expressed genes in the experimental data set versus (2) total number of genes associated with a defined biological process in the reference data set/total number of genes in the reference data set. Information about subcellular localization of differentially expressed transcripts was taken from the COMPARTMENTS database [51].

### Preparation of mouse heart mitochondria

Animals were killed by exsanguination under deep anesthesia using Ketamine/Xylazine (12/1.6 mg/kg body weight). Hearts were prepared after perfusion with ice-cold PBS and cut into halves. The halves were snap frozen in liquid nitrogen and stored at −80°C. After thawing, intact heart mitochondria were prepared as described earlier for mitochondria from rat organs [52]. Buffer volumes were reduced by a factor of approximately 2 to account for the lower organ size in mice. In detail, fat, clotted blood, auricles, and fasciae were removed from dry hearts. Hearts were cut into 1–2 mm pieces. Pieces were collected in 10 ml of washing buffer (0.3 M sucrose, 10 mM HEPES pH 7.2, 0.2 mM EDTA), 250 μl Trypsin (bovine pancreas type I, Sigma) of a 2.5 mg/ml stock solution was added, and minced tissue was further homogenized with an Ultra Turrax (IKA-TIO Basic; 3 × 5 seconds). After constant stirring for 15 minutes, 5 ml of mitochondria isolation buffer (20 mM HEPES, pH 7.4, 10 mM KCl, 5 mM MgCl_2_, 1 mM EDTA, 1 mM EGTA, 250 mM sucrose) containing 3.25 mg Trypsin inhibitor (*Glycine max*, Sigma) was added. Samples were centrifuged for 10 minutes at 900 xg at 4°C to remove debris. The resulting supernatant was transferred to a fresh Eppendorf tube and centrifuged again for 15 minutes at 10,000 xg at 4°C. After centrifugation, the supernatant was discarded, and the pellet was rinsed twice with fresh mitochondrial isolation buffer, removing the fluffy white outer rim layer. The resulting brown pellet containing intact mitochondria was resuspended in mitochondria isolation buffer.

### Mitochondrial respiration

The rate of mitochondrial respiration was monitored at 25°C using an Oxygraph-*2k* system (Oroboros) equipped with 2 chambers and DatLab software as previously described, with slight modifications [53]. In detail, 200–300 μg of heart mitochondria were added to 2 ml of a buffer containing 200 mM sucrose, 10 mM potassium phosphate, 0.1% bovine serum albumin, 10 mM Tris-HCl, 10 mM MgSO_4_, and 2 mM EDTA, pH 7.0; and respiration was measured. Oxygen consumption was measured after the addition of the NADH-generating substrates malate (0.5 mM) and glutamate (0.5 mM). Then, ADP (0.15 mM) was added. To inhibit complex I activity, rotenone was added to a final concentration of 100 nM. Then, succinate (10 mM) was added, and complex II–dependent respiration was determined. Finally, KCN (2 mM) was added to inhibit complex IV activity.

Heart mitochondria from p27-deficient and wild-type littermates were always measured blinded in parallel, using the same conditions. The same setup was applied to measure respiration of heart mitochondria isolated from mice that had received caffeine with the drinking water or water. For each preparation, a second set of measurements was performed in a crossover design.

### Diabetogenic diet

Male mice at the age of 7–8 weeks were fed a diabetogenic diet (S7200-E010, EF Bio-Serv F1850mod; containing 24% sucrose, 35.85% lard, Ssniff) for 9.5 weeks, leading to a prediabetic state and increased body weight gain. After that, animals were randomized to a control group (diabetogenic diet) or a group receiving additional 0.05% caffeine in the drinking water (diabetogenic diet + caffeine) 10 days prior to ischemia induction. Caffeine treatment was continued until the end of the experiment 3 weeks post ischemia. The composition of the diabetogenic diet is detailed in S2 Table.

### Myocardial ischemia and reperfusion

A closed-chest model of reperfused myocardial infarction was utilized. Mice were anesthetized by intraperitoneal injection of ketamine (100 mg/kg body weight) and xylazine (10 mg/kg body weight), intubated, and ventilated with a tidal volume of 10 μl/g body weight at a rate of 140 strokes/minute (two-thirds air, one-third oxygen and isoflurane 2.0 vol.% [Forene, Abbott GmbH]). Mice were placed in a supine position on a 38°C warmed plate to maintain body temperature. After a left lateral thoracotomy between the third and four rib, the pericardium was dissected, and a 7–0 surgical suture was passed underneath the left anterior descending coronary artery (LAD). Both ends of the surgical suture were threaded through a 1-mm section of PE-20 tubing, forming a loose snare around the LAD, and were exteriorized to the left side of the thorax. The suture was left in the subcutaneous tissue. At 3 days postinstrumentation, the animals were reanesthetized by mask inhalation of isoflurane 2.0 vol.% and a mixture of one-third oxygen and two-thirds room air. Mice were placed in a supine position on a 38°C warmed plate to maintain body temperature. The skin was reopened, and after dissecting the loop, both ends of the applied suture were gently pulled tight until ST-elevation appeared on the ECG. After 60 minutes of ischemia, reperfusion was accomplished by cutting the suture close to the chest wall. Reperfusion was confirmed by reduction of ST-elevation. Reperfusion was performed for 21 days. We strictly adhered to ischemia induction between 8 AM and 11 AM to ensure equal ischemia and reperfusion tolerance.

### Wall thickness and scar size determination

Three weeks post ischemia and reperfusion, animals were killed by CO_2_, and hearts were excised and rinsed in PBS. After dehydration, hearts preserved in Roti-Histofix 4% (Carl Roth) for 24 hours were paraffin-embedded and cut into 5-μm sections in 10 levels (approximately 100 µm) beginning from the apex up to the ligation side, discarding 250 μm between each level. To calculate scar size, fibrous area, and wall thickness, sections were stained with Gomori’s one-step trichrome staining. The heat-fixed sections were deparaffinized twice in Roti-Clear (Carl Roth) for 15 minutes and then rehydrated with a graded ethanol series to dH_2_O. Sections were incubated in Bouin’s solution (Sigma Aldrich) at 58°C for 15 minutes. After 5 minutes of rinsing under running water, nuclear staining was performed with Weigert's iron hematoxylin A and B for 5 minutes (1:1, Sigma Aldrich). The sections were rinsed again for 5 minutes with running water followed by 25 minutes of incubation with Gomori’s staining solution (chromotrope 2R, methylenblue, glacial acetic acid, phosphotungstic acid). Sections were briefly rinsed with water and 0.5% acetic acid 2x 2 minutes. Then sections were treated with an ascending alcohol series and Roti-Clear (2 x 5 minutes) and covered with Roti-Mount mounting medium (Carl Roth). Images were taken with a Zeiss Axio Imager M2. The circumference of the entire endocardium and epicardium and the thickness and length of the infarcted portion, fibrous area, and the left ventricle cavity area were determined using Diskus View software (Hilgers). Setting of the myocardial infarctions and scoring of scar size and left ventricular wall thickness were conducted in a blinded fashion and confirmed by an independent blinded observer.

### Immunostaining of heart slices

The sections were stained with antibodies against p27 (polyclonal, PA5-27188, Thermo Fisher Scientific, 1:25) and TIM23 (clone 32, BD Biosciences, 1:100). The sections were deparaffinized with xylene and rehydrated by a descending alcohol series. For p27 and TIM23, a basic target retrieval solution boiled for 20 minutes in Tris/EDTA buffer pH 9.0 (Dako) was required. The sections were cooled down, washed with PBS, and incubated with 4% formalin for 20 minutes in a wet chamber. Then, slices were rinsed with PBS and were treated with blocking solution Tris Buffered Saline (50 mM Tris-HCl, 150 mM NaCl, 2.5 mM KCl, pH 8.0) supplemented with 10% fetal bovine serum/3% goat serum/0.1%Triton-X 100 for 1 hour in a wet chamber. The sections were incubated with the primary antibodies overnight at 4°C in a wet chamber. Next, the slices were washed with PBS; the incubation with the respective secondary antibodies (anti-rabbit IgG (H + L) cross-absorbed antibody, Alexa Fluor 647, A21244, Thermo Fisher Scientific, 1:200; and anti-mouse IgG (H + L) cross-absorbed antibody, Alexa Fluor 568, A11004, Thermo Fisher Scientific, 1:200) was performed for 1 hour in a wet chamber. The sections were covered with ProLong Diamond antifade mounting medium with DAPI (Invitrogen). Fluorescence images were taken with a Zeiss Axio Imager M2.

### Statistics

The number of experiments (*n*) given in the figure legends represents independent biological replicates. Normal distribution for all data sets was confirmed by Shapiro-Wilk test; homogeneity of variances (from means) between groups was verified by Levene’s test. Pairwise comparisons were performed with two-sided, unpaired Student *t* tests on raw data. Multiple comparisons were performed using one-way ANOVA with post-hoc Tukey HSD test. Sample sizes for experiments, which were based on the respective statistical tests for data analyses, were calculated employing G*Power version 3.1.9.2 [54]. Effect strength for this power calculation was taken from our earlier studies [8,44,53]. Significance level (α-error) and sensitivity (β-error) were set to 0.05 and 0.95, respectively.

## Supporting information

S1 Fig Caffeine induces migration despite specific adenosine receptor 2A or 2B inhibition. **(A)** A wound was set in a confluent monolayer of primary human endothelial cells, and the cells were treated with or without 50 μM caffeine and/or 100 nM SCH442416, a specific adenosine 2A receptor inhibitor, for 18 hours. Migratory capacity was assessed by counting cells migrated into the wound, using Image J. Data are mean ± SEM, *n* = 5–6, *p < 0.05 versus untreated, ^#^*p* < 0.05 versus SCH442416 (one-way ANOVA). **(B)** A wound was set, and cells were treated with or without 50 μM caffeine and/or 100 nM GS6201, a specific adenosine 2B receptor inhibitor, for 18 hours. Migratory capacity was assessed by counting cells migrated into the wound, using Image J. Data are mean ± SEM, *n* = 6–7, *p < 0.05 versus untreated, ^#^*p* < 0.05 versus GS6201 (one-way ANOVA). Underlying data are provided in S1 Data. n.s., not significant.
(TIF)

S2 Fig Caffeine does not induce phosphorylation of PDE4A and PDE5A. Endothelial cells were treated with 50 μM caffeine for 18 hours, and PDE4A P-S686/688 and PDE5A P-S102, as well as total PDE4A and PDE5A, were detected by immunoblot. **(A)** Shown are 3 independent biological replicates for PDE4A P-S686/688 and PDE4A with the corresponding loading controls (Tubulin). **(B)** Semiquantitative analyses of the ratios of phospho PDE4A to total PDE4A. Data are mean ± SEM, *n* = 5 (two-tailed unpaired *t* test). **(C)** Shown are 3 independent biological replicates for PDE5A P-S102 and PDE5A with the corresponding loading controls (Tubulin). **(D)** Semiquantitative analyses of the ratios of phospho PDE5A to total PDE45A. Data are mean ± SEM, *n* = 5 (two-tailed unpaired t-test). Underlying data are provided in S1 Data. n.s., not significant; PDE4A, phosphodiesterase 4A; PDE4A P-S686/688, phosphorylation of serine 686 and 688 in PDE4A; PDE5A, phosphodiesterase 5A; PDE5A P-S102, phosphorylation of serine 102 in PDE5A.
(TIF)

S3 Fig Original blots used for the quantitation of the siRNA-mediated p27 knockdown. p27 was knocked down in endothelial cells by transfection with 2 different siRNAs targeting the p27 mRNA (p27 siRNA-1, p27 siRNA-2) or a scrambled siRNA (“scr”) as control, and p27 levels were determined by immunoblot. Shown are the blots for the 5 biological replicates used for the quantitation shown in Fig 1B. The levels of p27 were normalized to actin or tubulin, respectively. siRNA, small interfering RNA.
(TIF)

S4 Fig siRNA-mediated knockdown of p27 does not affect cellular and mitochondrial morphology. p27 was knocked down in endothelial cells by transfection with 2 different siRNAs targeting the p27 mRNA (siRNA p27-1, siRNA p27-2) or a scrambled siRNA (“scr”) as control. Intact cell morphology is shown in the brightfield images. To show the mitochondrial network and p27 distribution and levels, nuclei were visualized with DAPI (blue), mitochondria by staining for TIM23 (red), and p27 with a p27 antibody (green). Merge shows an overlay of all fluorescence channels. DAPI, 4′,6-diamidino-2-phenylindole; siRNA, small interfering RNA; TIM23, translocase of inner mitochondrial membrane 23.
(TIF)

S5 Fig Original blots used for the quantitation of the caffeine-induced mitochondrial translocation of p27. Endothelial cells were treated with 50 μM caffeine for 18 hours, and mitochondrial (“mito”) and nonmitochondrial (“non-mito”) fractions were separated. p27 levels in the mitochondrial fractions were determined by immunoblot and normalized to TIM23. Shown are the blots for the 6 biological replicates used for the quantitation shown in Fig 2B. TIM23, translocase of inner mitochondrial membrane 23.
(TIF)

S6 Fig Caffeine improves respiratory capacity and increases mitochondrial p27 in old mice to the level of adult mice. **(A)** For better comparability, the data for malate/glutamate- (“M/G”) and ADP-stimulated respiration of the mitochondria from the hearts of adult wild-type (“adult wt”) and p27-deficient (“adult p27ko”) mice from Fig 5B were combined with the data from the mitochondria from 22-month-old wild-type mice receiving water (“old wt”) or water with caffeine (“old wt+caffeine”) shown in Fig 8A. **(B)** Heart mitochondria from adult wild-type mice, old mice, and old mice that had received drinking water with 0.05% caffeine for 10 days were analyzed for mitochondrial p27 by immunoblot. To control for purity of the mitochondria, a total heart lysate (“lys”) was used in parallel, and Vimentin was detected. Underlying data are provided in S1 Data.
(TIF)

S7 Fig Digestion of mouse mitochondria with proteinase K. Forty μg of mouse mitochondria from old (22 months) and adult (6 months) mice as well as mice on a diabetogenic diet—presented in Figs 8C, 8E and 9E—were digested with proteinase K to obtain mitoblasts. Forty μg of undigested mitochondria and the resulting mitoblasts were loaded. Immunoblots for p27, TOM40, and TIM23 are shown. The absence of TOM40 and the presence TIM23 verify the proteinase K digest. TIM23, translocase of inner mitochondrial membrane 23; TOM40, translocase of outer mitochondrial membrane 40.
(TIF)

S1 Table GO terms for biological processes significantly (*p* < 0.05) enriched in hearts of wild-type mice after receiving 0.05% caffeine in the drinking water for 10 days compared to animals on drinking water alone, and subcellular localization of gene products. GO, gene ontology.
(XLSX)

S2 Table Composition of diabetogenic diet. (XLSX)

S1 Data Excel spreadsheet containing, in separate sheets, the underlying numerical data for figure panels 1B, 1C, 2B, 2F, 2G, 2H, 3D, 3E, 4B, 4E, 4G, 5A, 5B, 6B, 7C, 8A, 8B, 8D, 8F, 9B, 9C, 9F, S1A, S1B, S2B, S2D, and S6A. (XLSX)

## Abbreviations

αSMA: α smooth muscle actin; aa, amino acid
ADP: adenosine diphosphate
ATP: adenosine triphosphate
CDI: cyclin-dependent kinase inhibitor
CDK4: cyclin-dependent kinase 4
CDKN1A: cyclin-dependent kinase inhibitor 1A
CDKN1B: cyclin-dependent kinase inhibitor 1B
DAPI: 4′,6-diamidino-2-phenylindole
GO: gene ontology
GRP75: 75 KDa glucose-regulated protein
HPF: high power field
HSPA9: heat shock protein 70 kDa
LAD: left anterior descending coronary artery
PDE: phosphodiesterase
PI: propidium iodide
siRNA: small interfering RNA
TGFβ1: transforming growth factor β1
TIM23: translocase of inner mitochondrial membrane 23
TOM40: translocase of outer mitochondrial membrane 40
Trx-1: thioredoxin-1
