# Supplementary figures and images for: CDKN1B/p27 is localized in mitochondria and improves respiration-dependent processes in the cardiovascular system—New mode of action for caffeine
Source: PLoS Biol. 2018 Jun 21;16(6):e2004408. doi: 10.1371/journal.pbio.2004408 (PMC6013014; doi:10.1371/journal.pbio.2004408)

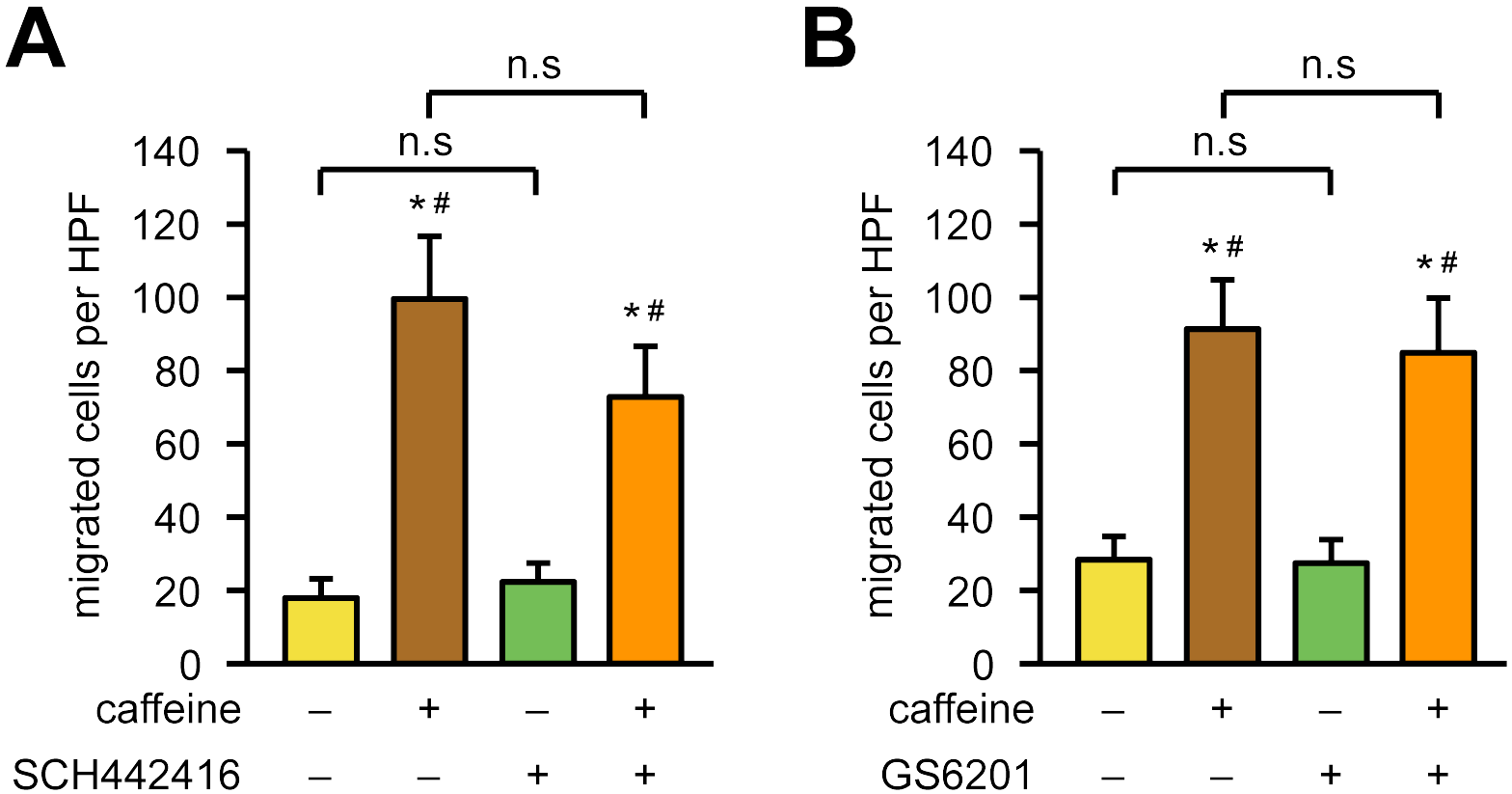

Supplement: S1 Fig — (A) A wound was set in a confluent monolayer of primary human endothelial cells, and the cells were treated with or without 50 μM caffeine and/or 100 nM SCH442416, a specific adenosine 2A receptor inhibitor, for 18 hours. Migratory capacity was assessed by counting cells migrated into the wound, using Image J. Data are mean ± SEM, n = 5–6, *p < 0.05 versus untreated, #p < 0.05 versus SCH442416 (one-way ANOVA). (B) A wound was set, and cells were treated with or without 50 μM caffeine and/or 100 nM GS6201, a specific adenosine 2B receptor inhibitor, for 18 hours. Migratory capacity was assessed by counting cells migrated into the wound, using Image J. Data are mean ± SEM, n = 6–7, *p < 0.05 versus untreated, #p < 0.05 versus GS6201 (one-way ANOVA). Underlying data are provided in S1 Data. n.s., not significant. (TIF) [file pbio.2004408.s001.tif]

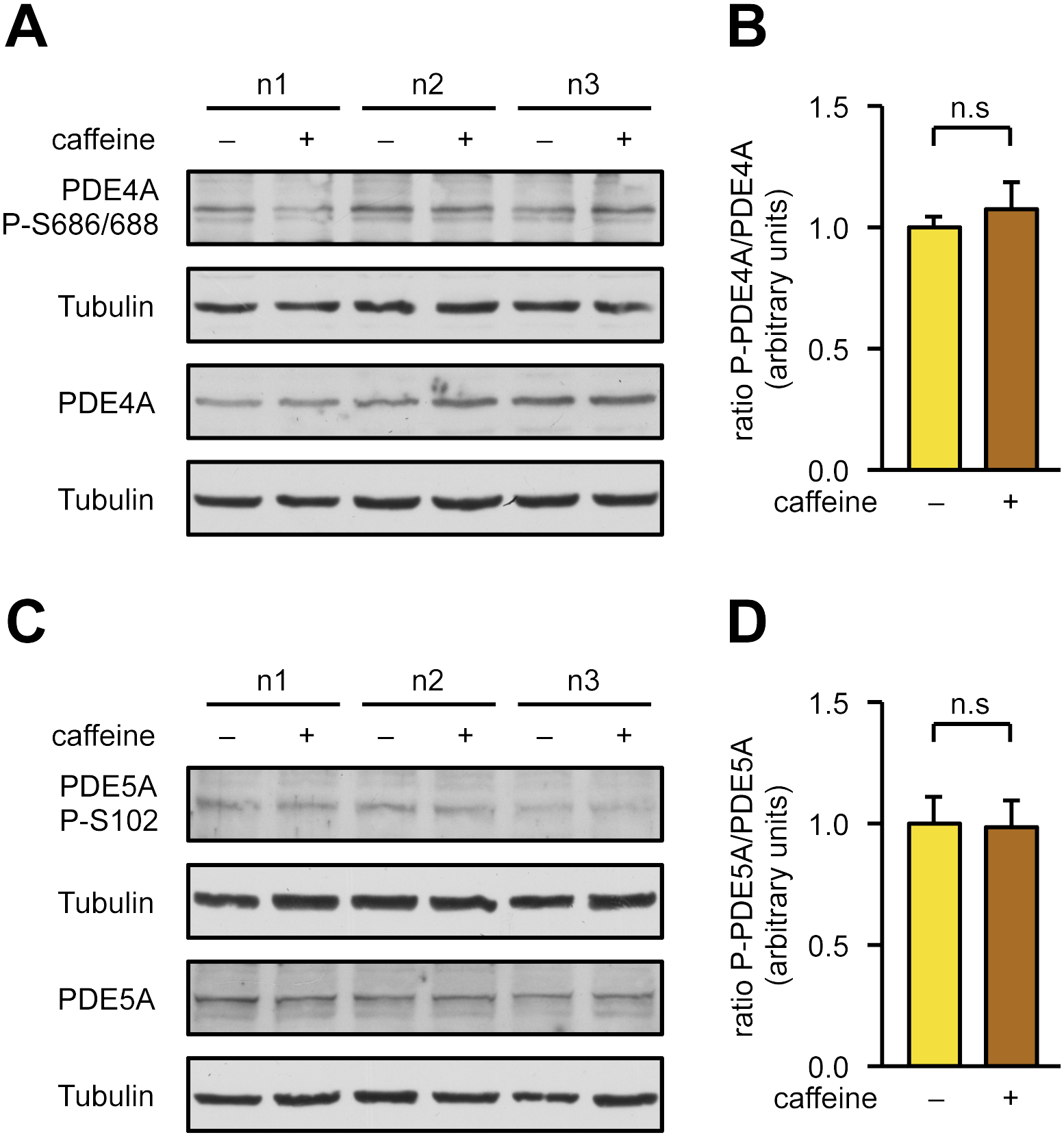

Supplement: S2 Fig — Endothelial cells were treated with 50 μM caffeine for 18 hours, and PDE4A P-S686/688 and PDE5A P-S102, as well as total PDE4A and PDE5A, were detected by immunoblot. (A) Shown are 3 independent biological replicates for PDE4A P-S686/688 and PDE4A with the corresponding loading controls (Tubulin). (B) Semiquantitative analyses of the ratios of phospho PDE4A to total PDE4A. Data are mean ± SEM, n = 5 (two-tailed unpaired t test). (C) Shown are 3 independent biological replicates for PDE5A P-S102 and PDE5A with the corresponding loading controls (Tubulin). (D) Semiquantitative analyses of the ratios of phospho PDE5A to total PDE45A. Data are mean ± SEM, n = 5 (two-tailed unpaired t-test). Underlying data are provided in S1 Data. n.s., not significant; PDE4A, phosphodiesterase 4A; PDE4A P-S686/688, phosphorylation of serine 686 and 688 in PDE4A; PDE5A, phosphodiesterase 5A; PDE5A P-S102, phosphorylation of serine 102 in PDE5A. (TIF) [file pbio.2004408.s002.tif]

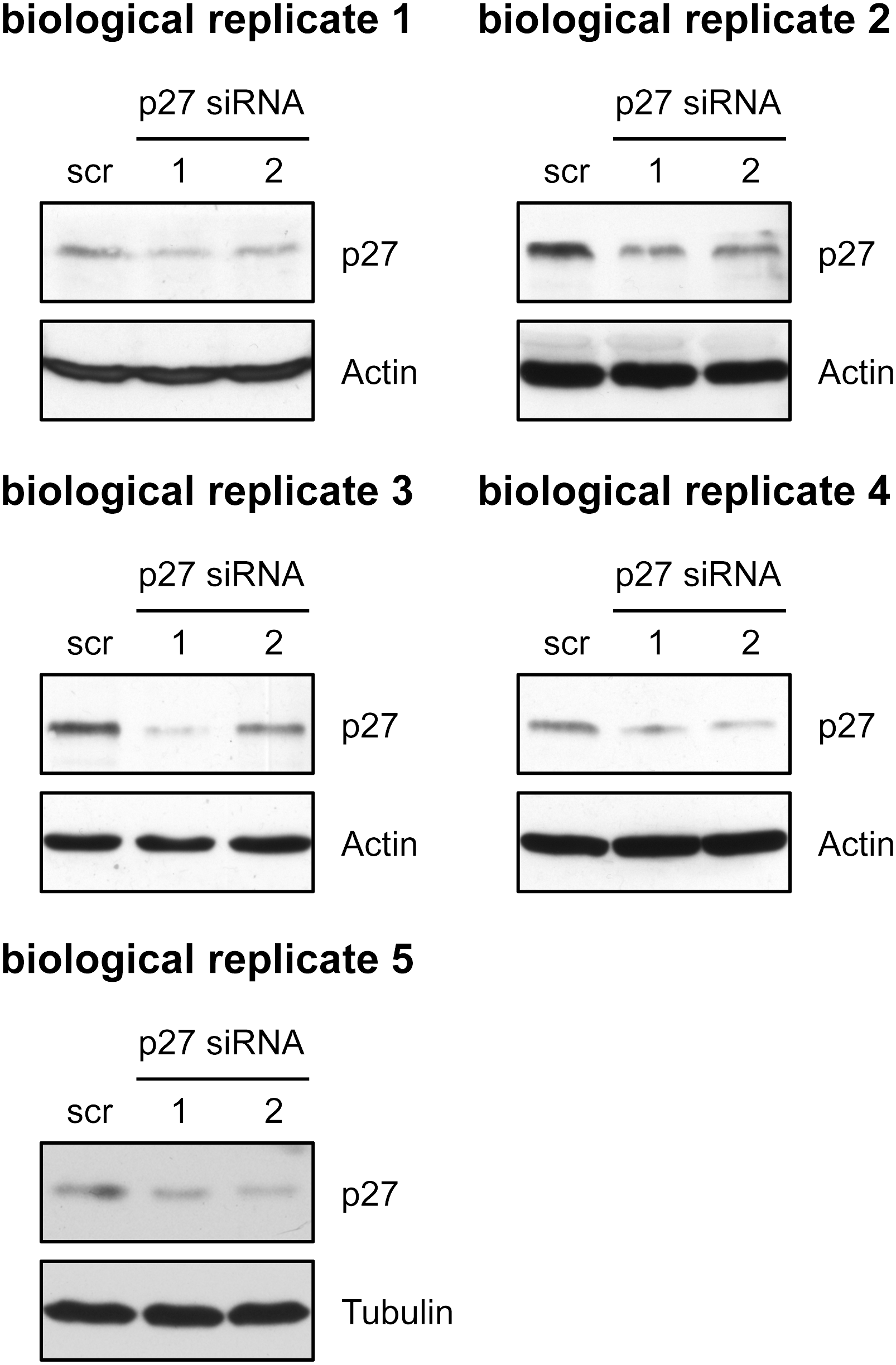

Supplement: S3 Fig — p27 was knocked down in endothelial cells by transfection with 2 different siRNAs targeting the p27 mRNA (p27 siRNA-1, p27 siRNA-2) or a scrambled siRNA (“scr”) as control, and p27 levels were determined by immunoblot. Shown are the blots for the 5 biological replicates used for the quantitation shown in Fig 1B. The levels of p27 were normalized to actin or tubulin, respectively. siRNA, small interfering RNA. (TIF) [file pbio.2004408.s003.tif]

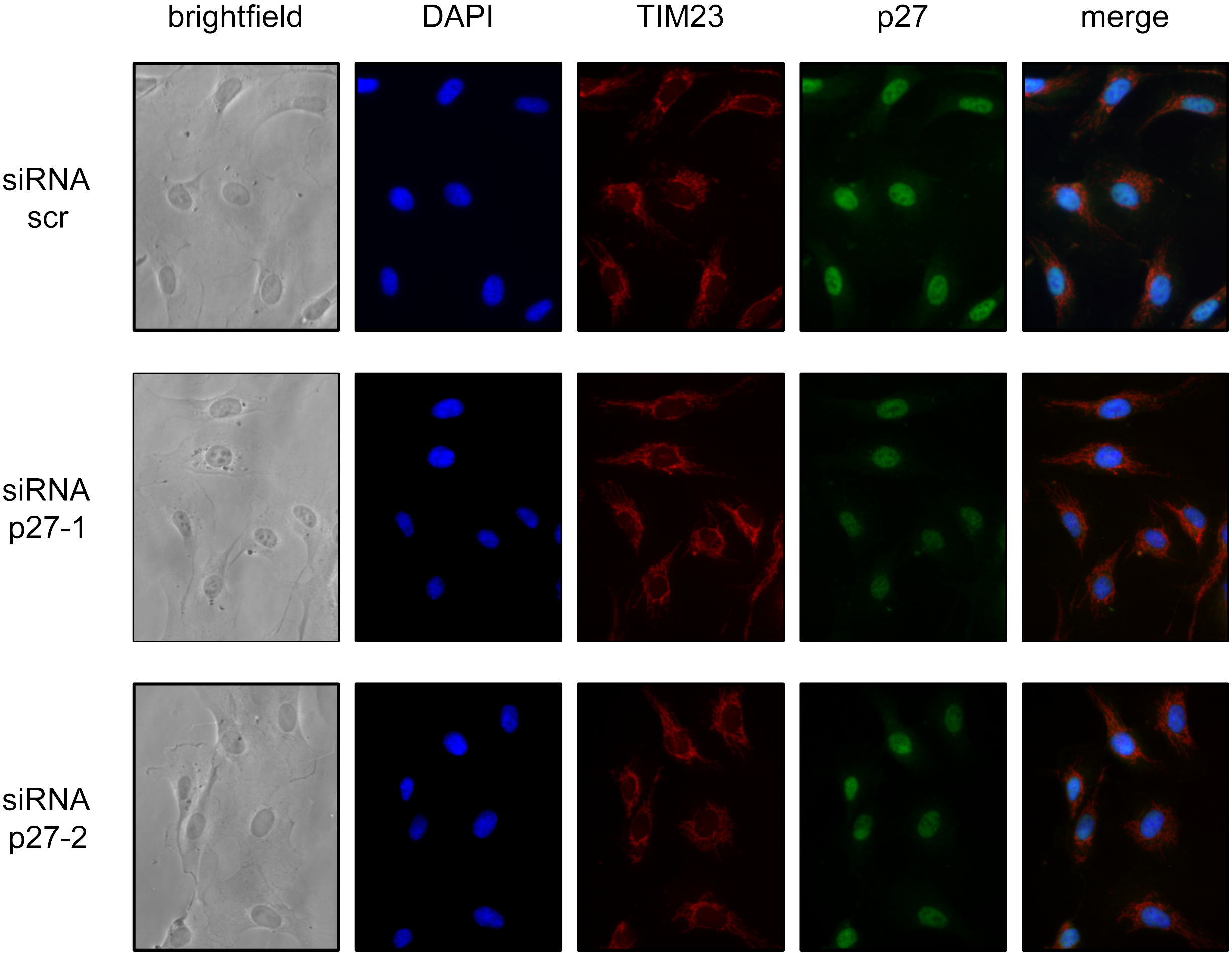

Supplement: S4 Fig — p27 was knocked down in endothelial cells by transfection with 2 different siRNAs targeting the p27 mRNA (siRNA p27-1, siRNA p27-2) or a scrambled siRNA (“scr”) as control. Intact cell morphology is shown in the brightfield images. To show the mitochondrial network and p27 distribution and levels, nuclei were visualized with DAPI (blue), mitochondria by staining for TIM23 (red), and p27 with a p27 antibody (green). Merge shows an overlay of all fluorescence channels. DAPI, 4′,6-diamidino-2-phenylindole; siRNA, small interfering RNA; TIM23, translocase of inner mitochondrial membrane 23. (TIF) [file pbio.2004408.s004.tif]

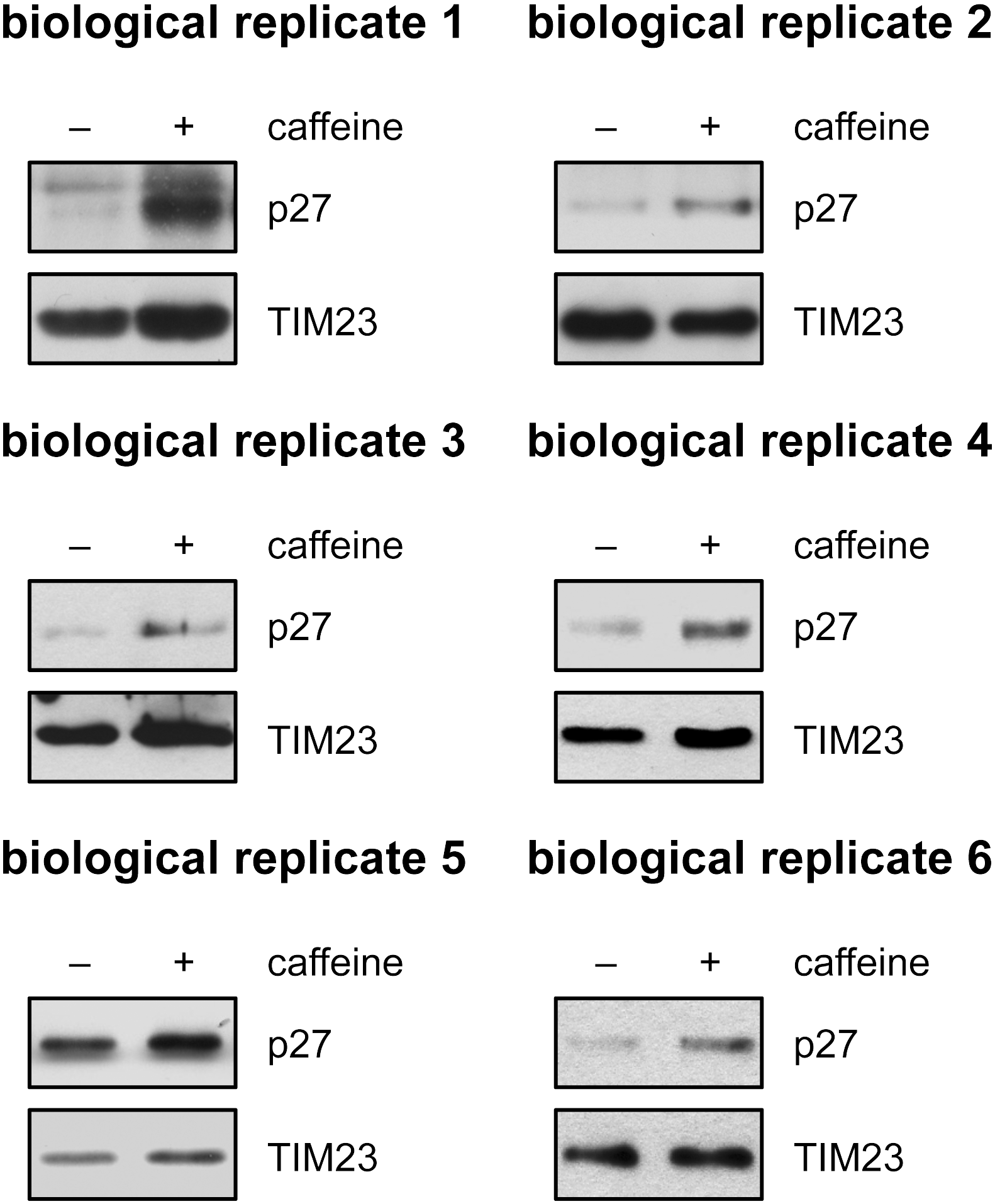

Supplement: S5 Fig — Endothelial cells were treated with 50 μM caffeine for 18 hours, and mitochondrial (“mito”) and nonmitochondrial (“non-mito”) fractions were separated. p27 levels in the mitochondrial fractions were determined by immunoblot and normalized to TIM23. Shown are the blots for the 6 biological replicates used for the quantitation shown in Fig 2B. TIM23, translocase of inner mitochondrial membrane 23. (TIF) [file pbio.2004408.s005.tif]

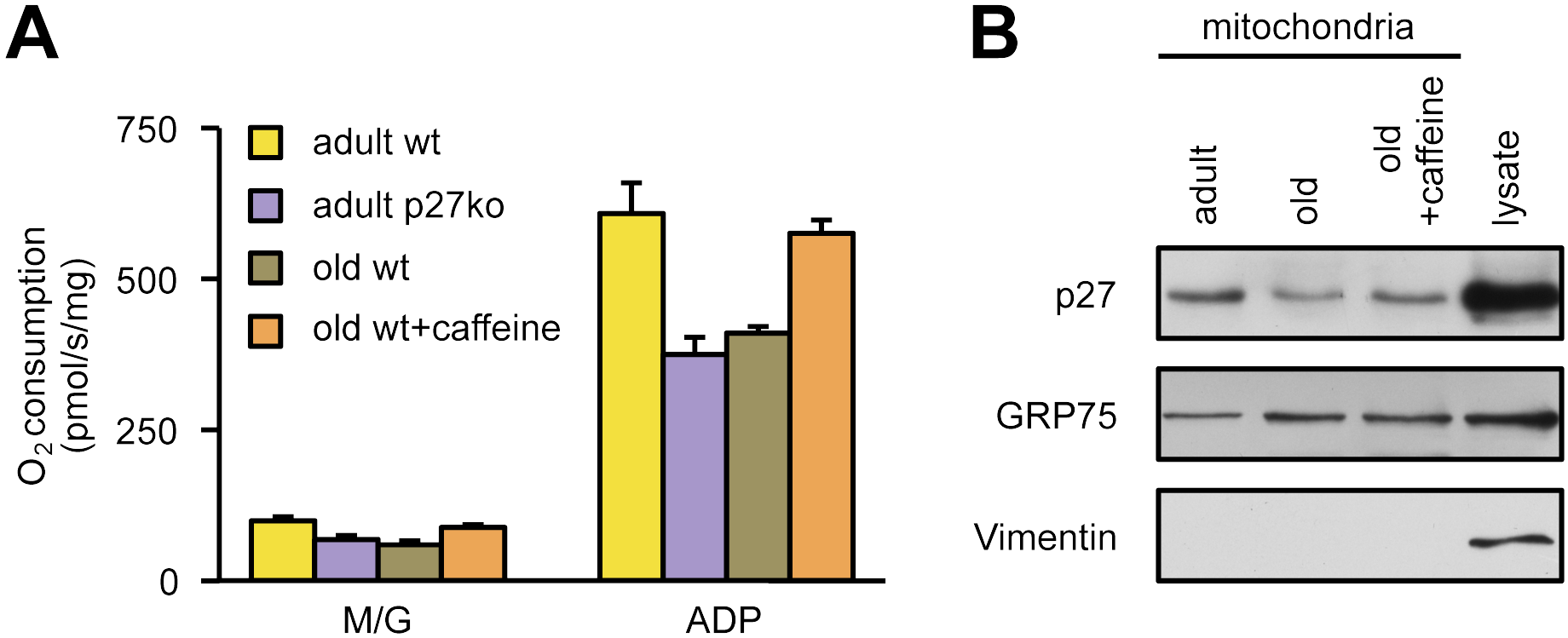

Supplement: S6 Fig — (A) For better comparability, the data for malate/glutamate- (“M/G”) and ADP-stimulated respiration of the mitochondria from the hearts of adult wild-type (“adult wt”) and p27-deficient (“adult p27ko”) mice from Fig 5B were combined with the data from the mitochondria from 22-month-old wild-type mice receiving water (“old wt”) or water with caffeine (“old wt+caffeine”) shown in Fig 8A. (B) Heart mitochondria from adult wild-type mice, old mice, and old mice that had received drinking water with 0.05% caffeine for 10 days were analyzed for mitochondrial p27 by immunoblot. To control for purity of the mitochondria, a total heart lysate (“lys”) was used in parallel, and Vimentin was detected. Underlying data are provided in S1 Data. (TIF) [file pbio.2004408.s006.tif]

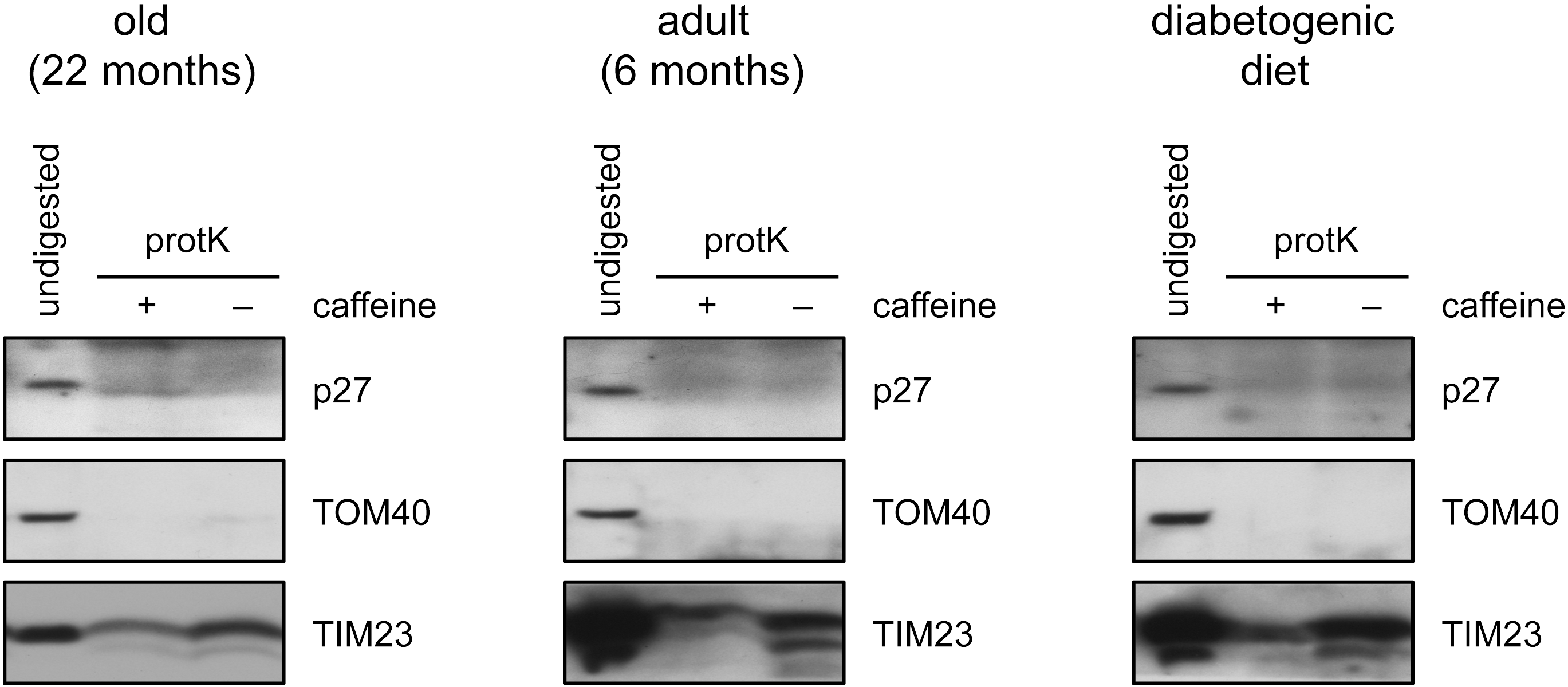

Supplement: S7 Fig — Forty μg of mouse mitochondria from old (22 months) and adult (6 months) mice as well as mice on a diabetogenic diet—presented in Figs 8C, 8E and 9E—were digested with proteinase K to obtain mitoblasts. Forty μg of undigested mitochondria and the resulting mitoblasts were loaded. Immunoblots for p27, TOM40, and TIM23 are shown. The absence of TOM40 and the presence TIM23 verify the proteinase K digest. TIM23, translocase of inner mitochondrial membrane 23; TOM40, translocase of outer mitochondrial membrane 40. (TIF) [file pbio.2004408.s007.tif]
